# Mathematical analysis of robustness of oscillations in models of the mammalian circadian clock

**DOI:** 10.1371/journal.pcbi.1008340

**Published:** 2022-03-18

**Authors:** Xiangyu Yao, Benjamin L. Heidebrecht, Jing Chen, John J. Tyson

**Affiliations:** 1 Graduate Program in Genetics, Bioinformatics and Computational Biology, Virginia Tech, Blacksburg, Virginia, United States of America; 2 Division of Systems Biology, Virginia Tech, Blacksburg, Virginia, United States of America; 3 Department of Biological Sciences, Virginia Tech, Blacksburg, Virginia, United States of America; 4 Fralin Life Sciences Institute, Virginia Tech, Blacksburg, Virginia, United States of America; University of California Irvine, UNITED STATES

## Abstract

Circadian rhythms in a wide range of organisms are mediated by molecular mechanisms based on transcription-translation feedback. In this paper, we use bifurcation theory to explore mathematical models of genetic oscillators, based on Kim & Forger’s interpretation of the circadian clock in mammals. At the core of their models is a negative feedback loop whereby PER proteins (PER1 and PER2) bind to and inhibit their transcriptional activator, BMAL1. For oscillations to occur, the dissociation constant of the PER:BMAL1 complex, ${\hat{K}}_{d}$, must be ≤ 0.04 nM, which is orders of magnitude smaller than a reasonable expectation of 1–10 nM for this protein complex. We relax this constraint by two modifications to Kim & Forger’s ‘single negative feedback’ (SNF) model: first, by introducing a multistep reaction chain for posttranscriptional modifications of *Per* mRNA and posttranslational phosphorylations of PER, and second, by replacing the first-order rate law for degradation of PER in the nucleus by a Michaelis-Menten rate law. These modifications increase the maximum allowable ${\hat{K}}_{d}$ to ~2 nM. In a third modification, we consider an alternative rate law for gene transcription to resolve an unrealistically large rate of *Per2* transcription at very low concentrations of BMAL1. Additionally, we studied extensions of the SNF model to include a second negative feedback loop (involving REV-ERB) and a supplementary positive feedback loop (involving ROR). Contrary to Kim & Forger’s observations of these extended models, we find that, with our modifications, the supplementary positive feedback loop makes the oscillations more robust than observed in the models with one or two negative feedback loops. However, all three models are similarly robust when accounting for circadian rhythms (~24 h period) with ${\hat{K}}_{d}$ ≥ 1 nM. Our results provide testable predictions for future experimental studies.

## Author summary

The circadian rhythm aligns bodily functions to the day/night cycle and is important for our health. The rhythm originates from an intracellular molecular clock mechanism that mediates rhythmic gene expression. It is long understood that transcriptional negative feedback with sufficient time delay is key to generating circadian oscillations. However, some of the most widely cited mathematical models for the circadian clock suffer from problems of parameter ‘fragilities’. That is, sustained oscillations are possible only for physically unrealistic parameter values. A recent model by Kim & Forger nicely incorporates the inhibitory binding of PER proteins to their transcription activator BMAL1, but oscillations in the Kim-Forger model require a binding affinity between PER and BMAL1 that is orders of magnitude larger than observed binding affinities of protein complexes. To rectify this problem, we make several physiologically credible modifications to the Kim-Forger model, which allow oscillations to occur with more realistic binding affinities. The modified model is further extended to explore the potential roles of supplementary feedback loops in the mammalian clock mechanism. Ultimately, accurate models of the circadian clock will provide better predictive tools for chronotherapy and chrono-pharmacology studies.

## Introduction

**Fig 1:**
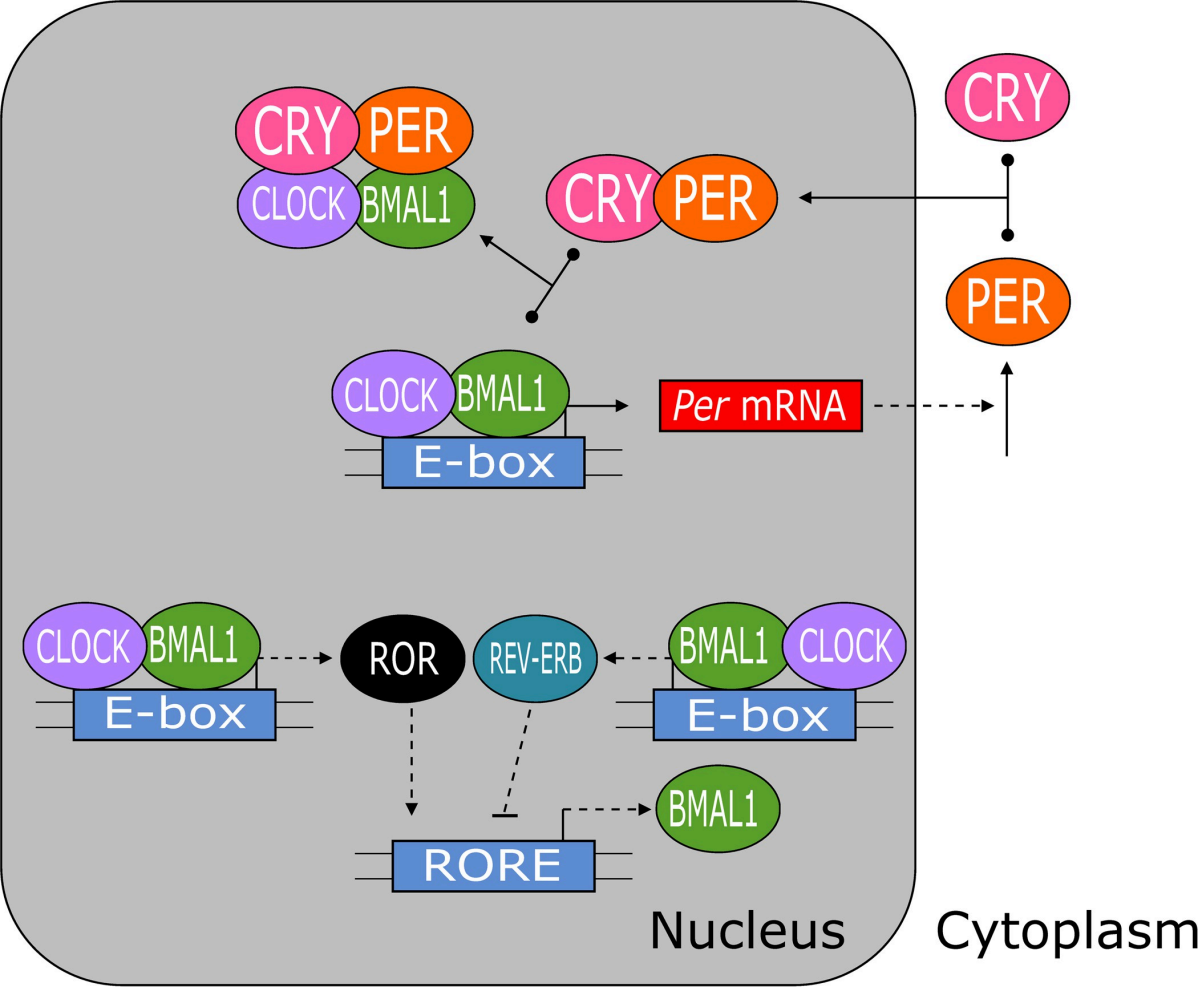
Three major feedback loops regulate the mammalian circadian clock. The core negative feedback loop is between PER:CRY and BMAL1:CLOCK. The two sources of additional feedback are negative feedback between REV-ERB and BMAL1 and positive feedback between ROR and BMAL1. PER1/2, CRY1/2, REV-ERBα/β and RORα/β are simplified to PER, CRY, REV-ERB and ROR, respectively. Solid lines indicate chemical reactions; the T-shaped reactions indicate reversible binding of proteins to form multicomponent complexes. Dashed lines indicate regulatory signals (positive regulation = barbed arrow, and negative regulation = blunt arrow).

Most organisms experience perpetual day/night cycles and need to synchronize their physiological functions with this potent external rhythm of light and temperature [1]. Endogenous circadian rhythms meet this demand. These autonomous clock-like rhythms are driven by molecular mechanisms that generate oscillations of ~24 h period through negative feedback on gene expression [1–3]. Although the genes and proteins constituting the circadian clocks in animals, plants and fungi are quite different, their essential interactions are remarkably similar. In all cases, the clock mechanism features a ‘core’ negative feedback loop: *A activates B activates C inhibits A*. In mammals, this loop consists of transcriptional regulation involving six genes: *Per1/2*, *Cry1/2*, *Bmal1*, and *Clock* [1–4]. For convenience, in this work we drop the distinction between the homologous pairs of proteins PER1/2 and CRY1/2. In this mechanism (Fig 1), the heterodimeric transcription factor BMAL1:CLOCK activates *Per* transcription. *Per* mRNA is then translated in the cytoplasm, where PER protein binds with CRY and enters the nucleus. PER:CRY then binds with BMAL1:CLOCK to block its activation of *Per* transcription. PER:CRY’s cycle of production, nuclear entry, auto-inhibition, and subsequent degradation is widely acknowledged to be the source of circadian rhythmicity [5].

Over the past 50 years, many people have proposed mathematical models of circadian rhythms [5–11]. In 1965, Brian Goodwin proposed a model of periodic enzyme synthesis based on negative feedback on gene expression [12,13]. At the time, Goodwin was not attending to circadian rhythms, because nothing was known then about the negative feedback of PER on its own synthesis. But his model was picked up later by Peter Ruoff [14–18] to explain many characteristic features of circadian rhythms. Recently, the core negative feedback loop of Goodwin’s model was extended with other feedback loops (as in Fig 1) to create more comprehensive and realistic models of circadian rhythms [19–21]. While all of these models have much to commend, they suffer from a technical problem with the underlying ‘Goodwin oscillator’.

In his model of periodic enzyme synthesis, Goodwin assumed that the end-product of a metabolic pathway functioned as an inhibitor of expression of the gene encoding the first enzyme in the pathway. The inhibition was carried out by *p* molecules of the end-product binding cooperatively to the transcription factor for the gene. In this scenario the rate of transcription is given by a Hill function, $\frac{\alpha_{1}K^{p}}{{K^{p}+Z}^{p}}$, where *Z* = concentration of end-product, *α*_1_ = maximum rate of transcription, and *K* = end-product concentration at half-maximal rate of transcription. In S1 Text, we define Goodwin’s model precisely, discuss its basic problem (for the model to oscillate, *p* must be greater than 8, which is unreasonable), and we describe two changes to Goodwin’s model that permit oscillations for smaller values of *p*.

One particularly interesting modification to Goodwin’s model was made by Jae Kyoung Kim and Daniel Forger [20], who replaced Goodwin’s view—of negative feedback by cooperative binding of a generic ‘repressor’ to a gene promoter—with their own model of stoichiometric binding of a repressor (PER:CRY) to an activator (BMAL1:CLOCK) of gene expression. Some characteristic features of the two models have been compared in [22,23]. In the following section, we describe the Kim-Forger model. Then, in the ‘Results’ section, we show that, like Goodwin’s model, the Kim-Forger model also has a ‘parameter fragility’ problem. This analysis frames our proposals for more robust and realistic mathematical models of circadian clocks.

### Kim & Forger’s model

**Fig 2:**
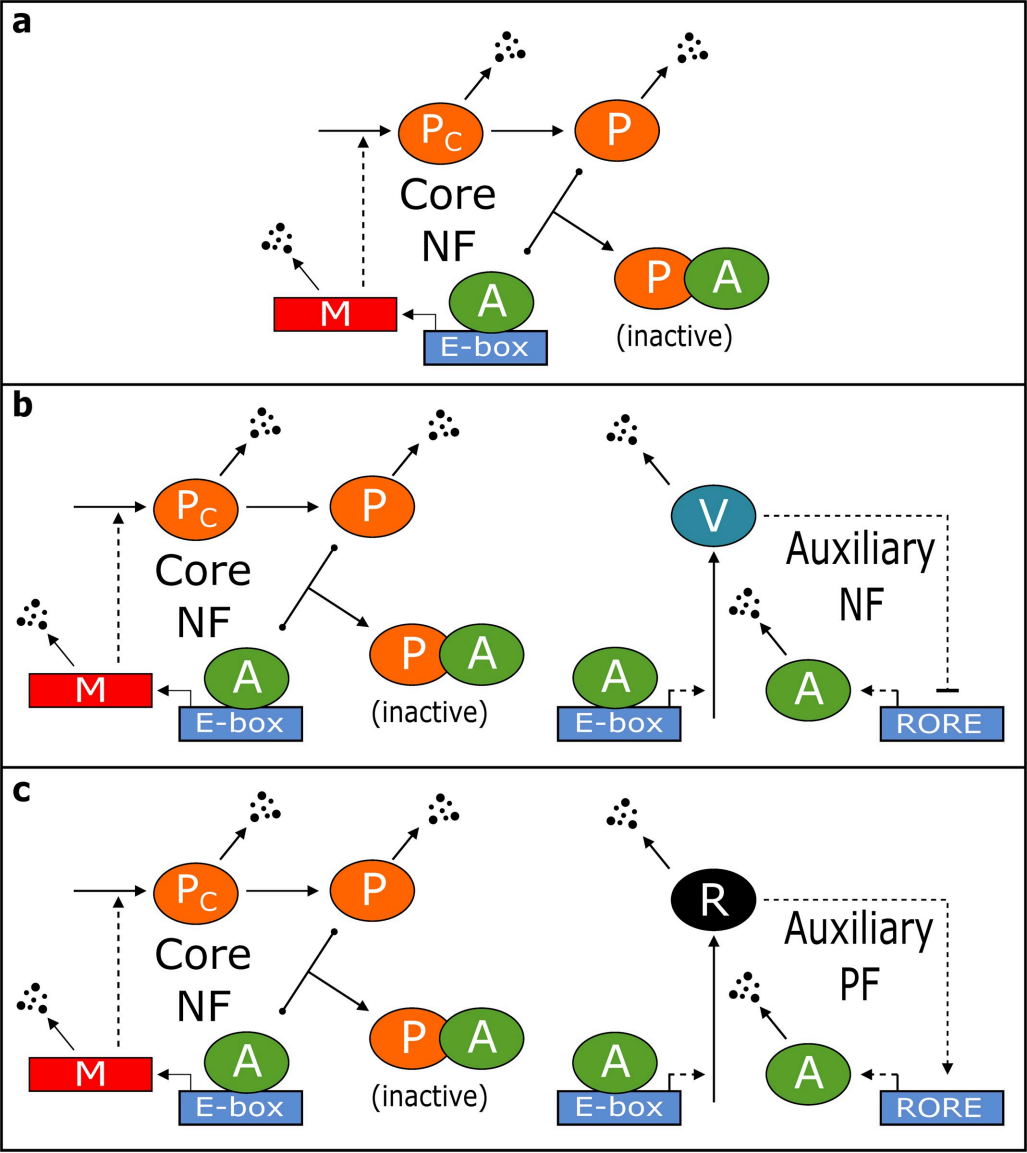
Wiring diagrams for the three Kim-Forger models: SNF (a), NNF (b), and PNF (c). To simplify the models, several molecular species that do not contribute significantly to the feedback loops are not explicitly represented. For example, in the SNF loop, CLOCK and CRY are not shown. In the NNF and PNF loops, mRNAs encoding REV-ERB, ROR and BMAL1 are not shown, nor are the cytoplasmic forms of these proteins. Solid and dashed lines indicate reactions and regulations, as in Fig 1. NF, negative feedback; PF, positive feedback.

In 2012, Kim & Forger [20] presented a model of the negative feedback loop generating autonomous circadian rhythms in mammalian cells (Fig 2A). The Kim-Forger (KF) ODEs are:

Kim-Forger SNF Model.


$$\frac{d\hat{M}}{d\hat{t}}={\hat{\alpha }}_{1}\frac{{\hat{A}}_{free}}{{\hat{A}}_{T}}-{\hat{\beta }}_{1}\hat{M}\;\frac{dM}{dt}=\alpha \frac{A_{free}}{A_{T}}-M$$ (1)

$$\frac{d{\hat{P}}_{c}}{d\hat{t}}={\hat{\alpha }}_{2}\hat{M}-{{\hat{\beta }}_{2}\hat{P}}_{c}\;\frac{dP_{c}}{dt}=M-P_{c}$$ (2)

$$\frac{d\hat{P}}{d\hat{t}}={\hat{\alpha }}_{3}{\hat{P}}_{c}-{\hat{\beta }}_{3}\hat{P}\;\frac{dP}{dt}=P_{c}-P$$ (3)

$${\hat{A}}_{\mathrm{free}}=\frac{1}{2}\left[{\hat{A}}_{T}-\hat{P}-{\hat{K}}_{d}+\sqrt{{\left({\hat{A}}_{T}-\hat{P}-{\hat{K}}_{d}\right)}^{2}+4{\hat{K}}_{d}{\hat{A}}_{T}}\right]$$

$$A_{\mathrm{free}}=\frac{1}{2}[A_{T}-P-1+\sqrt{{\left(A_{T}-P-1\right)}^{2}+4A_{T}}]$$ (4)

SNF stands for ‘single negative feedback’ (i.e., the core negative feedback loop involving PER:CRY inhibition of BMAL1:CLOCK). As originally written, the KF model has three dynamical variables: $\hat{M}$ = [*Per* mRNA], ${\hat{P}}_{c}$ = [PER protein in the cytoplasm], $\hat{P}$ = [PER protein in the nucleus] (i.e., PER:CRY in the nucleus). The BMAL1:CLOCK transcription factor is denoted by A; ${\hat{A}}_{T}$ is the total concentration of BMAL1:CLOCK in the nucleus, and ${\hat{A}}_{\mathrm{free}}$ is the concentration of ‘free’ BMAL1:CLOCK (i.e., not bound to PER:CRY) in the nucleus. (The ‘hat’ on each variable indicates a concentration in nanomole/liter; and $\hat{t}$ is time in hours.) The factor ${\hat{A}}_{\mathrm{free}}/{\hat{A}}_{T}$ is the probability that BMAL1:CLOCK is not bound to its repressor, PER:CRY. By expressing the rate of transcription of *Per* mRNA to be proportional to ${\hat{A}}_{\mathrm{free}}/{\hat{A}}_{T}$, Kim & Forger are implicitly assuming that the total number of BMAL1:CLOCK dimers is large enough to saturate the E-boxes on *Per* genes, and that PER:CRY binds equally well to BMAL1:CLOCK dimers that are either bound or not bound to an E-box. Eq (4) is derived by solving the condition for equilibrium binding of BMAL1:CLOCK (A) and PER:CRY (P) to form an inactive complex (C); namely, ${\hat{K}}_{d}\hat{C}={\hat{A}}_{\mathrm{free}}∙{\hat{P}}_{\mathrm{free}}=({\hat{A}}_{T}-\hat{C})(\hat{P}-\hat{C})$. The $\hat{\alpha }$’s and $\hat{\beta }$’s are rate constants with appropriate units of concentration and time. It is commonplace in these models to assume that ${\hat{\beta }}_{1}={\hat{\beta }}_{2}={\hat{\beta }}_{3}$, because this condition is most conducive to oscillations.

First of all, we cast the equations on the left side of (1)–(3) into dimensionless form on the right side by defining dimensionless concentrations, $P=\hat{P}/{\hat{K}}_{d},\;P_{c}={\hat{\alpha }}_{3}{\hat{P}}_{c}/\hat{\beta }{\hat{K}}_{d},\;M={{\hat{\alpha }}_{2}\hat{\alpha }}_{3}\hat{M}/{\hat{\beta }}^{2}{\hat{K}}_{d},\;A=\hat{A}/{\hat{K}}_{d}$, and dimensionless time, $t=\hat{\beta }\hat{t}$. Furthermore, $\alpha ={\hat{\alpha }}_{1}{{\hat{\alpha }}_{2}\hat{\alpha }}_{3}/{\hat{\beta }}^{3}{\hat{K}}_{d}$ is the dimensionless rate of synthesis of *Per* mRNA (in a wild-type diploid cell). The other dimensionless parameter in Eqs (1)–(4) is $A_{T}={\hat{A}}_{T}/{\hat{K}}_{d}$ = total concentration of BMAL1 in the nucleus.

In addition to the SNF model, Kim & Forger proposed two extended models, in which the core negative feedback loop involving PER and BMAL1 is supplemented with either an additional negative feedback from REV-ERB on transcription of the *Bmal1* gene (called the NNF model, Fig 2B) or an additional positive feedback from ROR on transcription of the *Bmal1* gene (called the PNF model, Fig 2C). Evidences for these interactions are found in references [24–28]. The ODEs of Kim & Forger’s ‘NNF’ and ‘PNF’ models are presented in S2 Text.

Notice that, in the SNF model, nonlinearity in the transcription term is due to tight stoichiometric binding between PER and BMAL1, not (as in Goodwin’s equations) to cooperative participation of nuclear PER in the regulation of *Per* gene expression. Consequently, the SNF model circumvents the unreasonable cooperativity constraint (*p* > 8) of Goodwin’s model. (Don’t confuse the Hill exponent, *p*, in Goodwin’s model with the concentration of nuclear PER, *P*, in the KF model.)

While the SNF model appears to oscillate robustly and avoid Goodwin’s unrealistic constraint (*p* > 8), the SNF model has an unrealistic constraint of its own. To elaborate, we derive an equation for oscillations to arise in the SNF model.

## Results

### Locus of Hopf bifurcations in Kim & Forger’s SNF model

The condition for a Hopf bifurcation in Eqs (1)–(4) is

$$8=\frac{\alpha }{A_{T}}{\left|\frac{\partial A_{\mathrm{free}}}{\partial P}\right|}_{\mathrm{ss}}=\frac{\alpha }{{2A}_{T}}\left[1+\frac{A_{T}-P_{\mathrm{ss}}-1}{\sqrt{{\left(A_{T}-P_{\mathrm{ss}}-1\right)}^{2}+4A_{T}}}\right]$$ (5)

where *P*_ss_, the steady-state solution of Eqs (1)–(4), satisfies the equation

$$P_{\mathrm{ss}}=\frac{\alpha }{2A_{T}}[A_{T}-P_{\mathrm{ss}}-1+\sqrt{{\left(A_{T}-P_{\mathrm{ss}}-1\right)}^{2}+4A_{T}}]$$ (6)

Solving Eqs (5) and (6) simultaneously, we find that

$$8=\frac{P_{\mathrm{ss}}}{\left(1+2\frac{A_{T}}{\alpha })P_{\mathrm{ss}}-(A_{T}-1\right)}$$ (7)

and from Eq (6) we derive

$$P_{\mathrm{ss}}=\frac{\alpha (A_{T}-1)}{2(\alpha +A_{T})}\left[1+\sqrt{1+\frac{4(\alpha +A_{T})}{{\left(A_{T}-1\right)}^{2}}}\right]$$ (8)

Substituting (8) into (7), we find, after a little algebra, the condition for a Hopf bifurcation:

$$\Phi ∙\alpha^{2}-\Psi (A_{T})∙\alpha +\Omega (A_{T})=0$$ (9)

where

$$\Phi =49,\Psi (A_{T})=8(A_{T}^{2}-30A_{T}+1),\Omega (A_{T})=64A_{T}{\left(A_{T}+1\right)}^{2}$$ (10)

**Fig 3:**
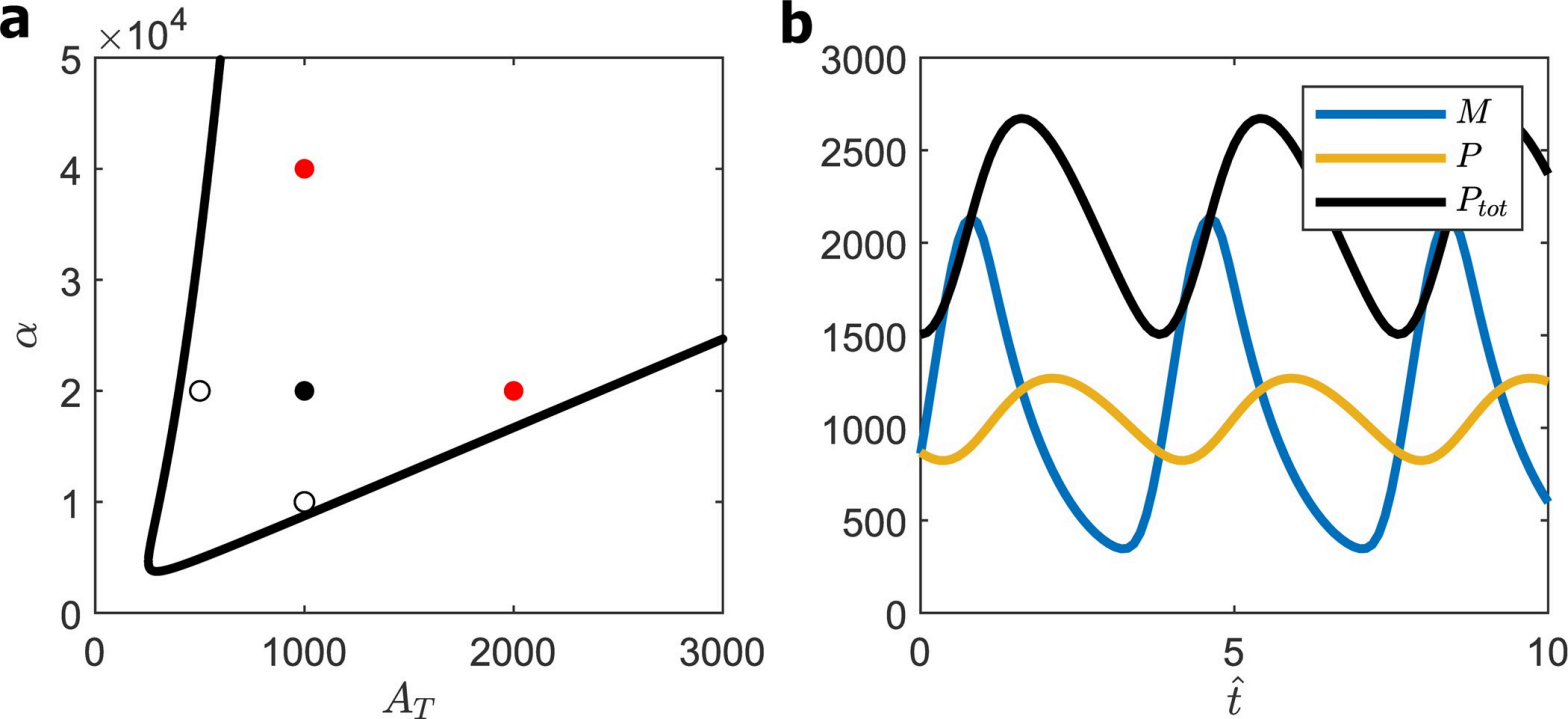
SNF (0L3) model. **(a)** Hopf bifurcation curve given by Eqs (9 and 10). ‘Five-point’ criterion: black circle, ‘homozygous diploid’ cell; white circles, heterozygous diploid cells; red circles, tetraploid cells. **(b)** Oscillations for homozygous diploid cells (*A*_T_ = 1000, *α* = 20,000), Period = 3.8; $\underset{t}{\max}\;P_{\mathrm{tot}}(t)$ = 2650.

Solving the quadratic Eq (9), we obtain *α* as a function of *A*_T_, as plotted in Fig 3A. We must locate a wild-type (WT) cell somewhere within the oscillatory domain, far enough from the HB locus so that mutant cells overexpressing or under-expressing BMAL1 and PER are still rhythmic. To this end, we propose the following ‘five-point criterion’ for choosing the values of *α* and *A*_T_ for a WT cell: if the point $\left(\alpha^{\mathrm{WT}},A_{T}^{\mathrm{WT}}\right)$ locates a WT cell on the bifurcation diagram, then the points $\left(\frac{1}{2}\alpha^{\mathrm{WT}},A_{T}^{\mathrm{WT}}),(\alpha^{\mathrm{WT}},\frac{1}{2}A_{T}^{\mathrm{WT}}),(2\alpha^{\mathrm{WT}},A_{T}^{\mathrm{WT}}),(\alpha^{\mathrm{WT}},2A_{T}^{\mathrm{WT}}\right)$ should also lie within the oscillatory domain. We introduce this constraint because: *Bmal1*^+/−^ and *Clock*^+/−^ cells, i.e., $\left(\alpha^{\mathrm{WT}},\frac{1}{2}A_{T}^{\mathrm{WT}}\right)$ are rhythmic [29,30]; *Per1*^−/−^*Per2*^+/+^ and *Per1*^+/+^*Per2*^−/−^ cells, i.e., $\left(\frac{1}{2}\alpha^{\mathrm{WT}},A_{T}^{\mathrm{WT}}\right)$ are rhythmic [31–33]; mouse embryonic fibroblasts (MEFs) retain rhythmicity when co-overexpressing both *Bmal1* and *Clock* up to at least four-fold, see Fig 3C of [34]; and MEFs carrying extra copies of *Per1* or *Per2*, driven by a *Per2*-promoter, also retain rhythmicity, see Fig 6 of [34]. We choose the smallest values of *α*, *A*_T_ that satisfy these requirements, i.e., $\alpha^{\mathrm{WT}}=2\times 10^{4},A_{T}^{\mathrm{WT}}=10^{3}$ (see Fig 3A), to minimize the dimensionless concentrations of PER and BMAL1 in order to maximize the value of ${\hat{K}}_{d}$ given observed values of $\hat{P}$ and $\hat{A}$.

The oscillatory solution of the SNF model for this set of parameter values is plotted in Fig 3B. The dimensionless period of oscillation is 3.8, which would correspond to a 24 h rhythm if $\hat{\beta }$ = 0.16 h^−1^. Nuclear PER, *P* (*t*), executes nearly sinusoidal oscillations around a mean value of 1000. The oscillations of *Per* mRNA, on the other hand, are slightly non-sinusoidal. This property of the model is not in contradiction to the evidently sinusoidal oscillations exhibited by luciferase ‘reporter’ genes driven by *Per2* promoters [35] because those observations were made on populations of cells, which, in reality, cannot be perfectly synchronized. In S1 Fig we show that the *Per* mRNA oscillations reported in Fig 3B, when averaged over a population of cells with a 10% dispersion of phase, appear perfectly sinusoidal.

The oscillations in Fig 3B require [PER]_nuclear_ ≈ [BMAL1]_total_ $\approx 10^{3}{\hat{K}}_{d}$, i.e., that the dissociation constant of the PER:BMAL1 complex is much smaller than the concentrations of the binding partners. To see why this is a problem, we must estimate ${\hat{K}}_{d}$ by fitting [PER] and [BMAL1] to experimental data.

### Estimation of ${\hat{P}}_{\mathrm{tot}},{\hat{A}}_{T},\;\mathrm{and}\;{\hat{K}}_{d}$ from experimental data

We can estimate ${\hat{K}}_{d}$ from the fact that there is a maximum of ~30,000 molecules of PER in a mammalian cell [36]; hence, ${3\times 10^{4}=V}_{N}\hat{P}+V_{C}{\hat{P}}_{c}=V_{N}{\hat{K}}_{d}P+V_{C}\frac{\hat{\beta }}{{\hat{\alpha }}_{3}}{{\hat{K}}_{d}P}_{c}$. In the SNF model, cytoplasmic PER is transported into the nucleus, so the rate at which PER molecules are lost from the cytoplasm, $\hat{\beta }{\hat{P}}_{c}∙V_{C}$, must equal the rate at which PER molecules are gained in the nucleus, ${\hat{\alpha }}_{3}{\hat{P}}_{c}∙V_{N}$, assuming that there is not significant degradation of PER in the cytoplasm (for an order-of-magnitude estimation, this is a reasonable simplifying assumption). In this case, $\frac{\hat{\beta }}{{\hat{\alpha }}_{3}}=\frac{V_{N}}{V_{C}}$, and $3\times 10^{4}=V_{N}{\hat{K}}_{d}(P+P_{c})$. From the simulation in Fig 3B, we find that *P*_tot_ = *P*+*P*_c_≈2500 at the peak of its oscillation, and from BioNumbers [37], we find that the volume of a typical mammalian cell nucleus is ~500 fL. Hence, ${\hat{K}}_{d}\approx \frac{1}{2500}(\frac{3\times 10^{4}}{500\mathrm{fL}})(\frac{10^{15}\mathrm{fL}/L}{6\times 10^{14}/\mathrm{nmol}})\approx \frac{100\mathrm{nM}}{2500}\approx 0.04\;\mathrm{nM}$. In this case, ${\hat{A}}_{T}$ ≈ 40 nM, and the total number of BMAL1 molecules in a nucleus of volume ~500 fL would be ~12,000. The observed number of BMAL1 molecules in a cell is ~25,000 [36], which is not too far off, considering that some fraction of BMAL1 molecules may not localize to the nucleus or act as functional transcription factors.

Is ${\hat{K}}_{d}=0.04\;\mathrm{nM}$ a reasonable estimate of the affinity of PER for BMAL1? We expect the time constant for dissociation of the PER:BMAL1 complex to be on the order of minutes (i.e., ${\hat{k}}_{\mathrm{unbind}}>10^{-3}\;s^{-1}$), because, if dissociation of the complex were slower, then the negative feedback of PER on BMAL1 would react sluggishly to changes in nuclear PER concentration. Furthermore, Eq (4) implies equilibrium of PER-BMAL1 binding and would not hold with a much slower dissociation constant. With this estimate of the dissociation rate constant, the binding constant for the complex would have to be ${\hat{k}}_{\mathrm{bind}}=\frac{{\hat{k}}_{\mathrm{unbind}}}{{\hat{K}}_{d}}>\frac{0.001s^{-1}}{0.04\mathrm{nM}}=0.02\;{\mathrm{nM}}^{-1}s^{-1}=2\times 10^{7}M^{-1}s^{-1}.$ However, protein-protein binding rate constants are typically on the order of 10^6^ M^−1^s^−1^ [38]. So, we estimate that a physically realistic, minimum value for the dissociation constant of the PER:BMAL1 complex is ${\hat{K}}_{d,\min}\approx 1\;\mathrm{nM}$, and we conclude that the dissociation constant used in the SNF model is unrealistically small by at least 25-fold.

Fribourgh et al. [39] recently studied the docking of PER2:CRY1/2 to the core PAS domain of BMAL1:CLOCK and measured ${\hat{K}}_{d}\approx 400\;\mathrm{nM}$. This estimate of ${\hat{K}}_{d}$ is likely too large because the authors used only partial protein sequences. Since the true value of ${\hat{K}}_{d}$ is likely between 1 and 100 nM, we will take ${\hat{K}}_{d}=10\;\mathrm{nM}$ as our benchmark.

To summarize, we find that circadian oscillations in KF’s original SNF model require a value of the PER:BMAL1 dissociation constant, ${\hat{K}}_{d}$ ≈ 0.04 nM (or smaller), that is 250-fold less than a realistic estimate of ${\hat{K}}_{d,\mathrm{est}}$ = 10 nM, and 25-fold less than the minimum value, ${\hat{K}}_{\mathrm{d,min}}$ = 1 nM.

In this work we consider some realistic changes to the SNF model that increase the maximum permissible value of ${\hat{K}}_{d}$ for oscillations. In the process, we come up with some other surprising reassessments of the KF model and its extensions.

### Longer feedback loop and saturating PER degradation increase the oscillatory robustness of the Kim-Forger SNF model

Our primary goal in modifying KF models is to alleviate the unreasonable constraint on ${\hat{K}}_{d}$, the dissociation constant of the PER:BMAL1 complex. To this end, we consider two changes to the SNF model: first, increasing the number of dynamical species in the PER-BMAL1 negative feedback loop, and second, introducing a Michaelis-Menten rate law for the degradation of nuclear PER. These same changes are known to increase the robustness of Goodwin’s model (as explained in S1 Text).

#### Longer feedback loop

In the SNF model, there is only one intermediate (P_c_) between *Per* mRNA (M) and nuclear PER protein (P). However, the primary gene transcript must be processed and exported to the cytoplasm, where it is translated into nascent PER protein. PER protein must be phosphorylated multiple times (PER has 10–20 phosphorylation sites [40,41]) and bound to CRY before it is transported into the nucleus. These steps insert a considerable time lag between *Per* gene transcription and the negative feedback on BMAL1 activity. To account for this time delay, we replace P_c_ in the SNF model by a sequence of species, P_1_, …, P_*J*_ (note that the first few intermediates could be mRNA species), to obtain the modified ODEs:

$$\frac{dM}{dt}=\alpha ∙\frac{A_{free}}{A_{T}}-M$$ (11)

$$\frac{dP_{1}}{dt}=M-P_{1}$$ (12)

$$\frac{dP_{j}}{dt}=P_{j-1}-P_{j},j=2,\ldots ,J$$ (13)

$$\frac{dP}{dt}=P_{J}-P$$ (14)

where *N* = *J*+2 is the total length of the negative feedback loop, and *A*_free_ is still given by Eq (4). This change lengthens the time between *Per* mRNA transcription and the negative feedback signal generated by nuclear PER and consequently increases the oscillatory potential of the negative feedback loop [42].

The longer feedback loop changes the condition for a Hopf bifurcation to arise in ODEs (11)-(14): the number ‘8’ on the left-hand-side of Eq (5) is replaced by the number $S_{N}={\left[sec(\frac{\pi }{N})\right]}^{N}$. Following the same derivation as before, we find that Eq (9) determines *α* as a function of *A*_T_ at the Hopf bifurcation, provided that

$$\Phi ={\left(S_{N}-1\right)}^{2},\Psi (A_{T})=S_{N}({\left(A_{T}+1\right)}^{2}-4S_{N}A_{T}),\Omega (A_{T})={S_{N}}^{2}A_{T}{\left(A_{T}+1\right)}^{2}$$ (10')

**Fig 4:**
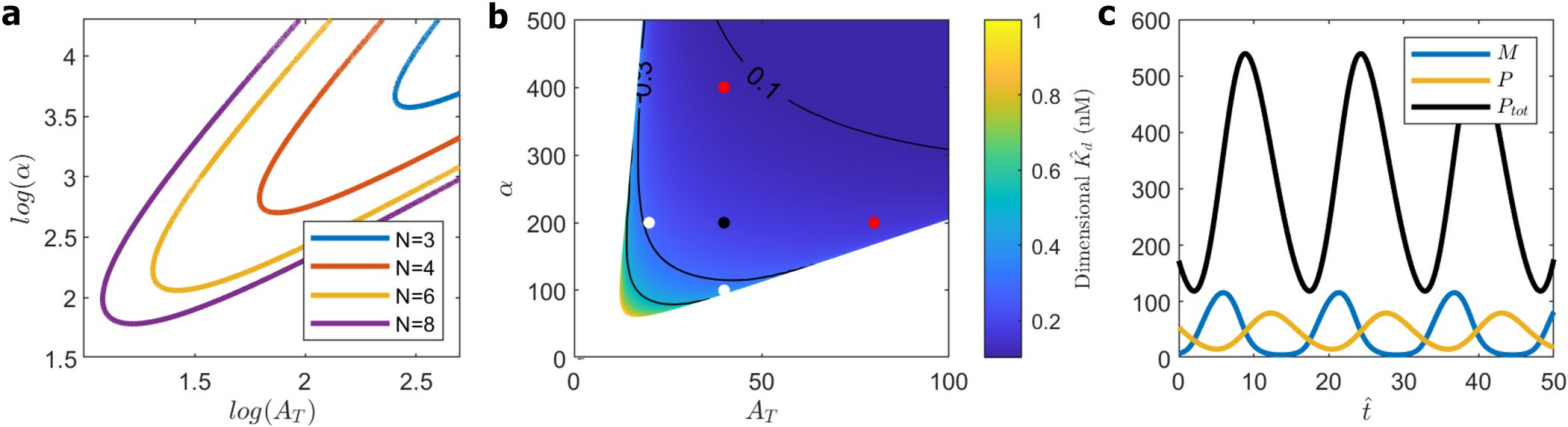
SNF (0LN) models. **(a)** Loci of Hopf bifurcations for *N* = 3, 4, 6, 8. **(b)** Value of ${\hat{K}}_{d}$ (expressed as 100nM/*P*_tot_) as a function of *A*_T_ and *α* for the SNF(0L8) model. Contour lines mark constant values of ${\hat{K}}_{d}$. Circles mark the ‘five-point’ criterion, as in Fig 3A. **(c)** Simulation of WT cell, SNF(0L8) with *A*_T_ = 40, *α* = 200. Period = 15.5, $\underset{t}{\max}\;P_{\mathrm{tot}}(t)$ = 540, ${\hat{K}}_{d}=0.2\;\mathrm{nM}$.

In Fig 4A we show that, as *N* (the length of the feedback loop) increases, the domain of oscillations in the (*α*, *A*_T_) parameter plane moves toward smaller values of *α* and *A*_T_. For example, for *N* = 8, applying the five-point criterion, we place the WT cell at *α* = 200, *A*_T_ = 40; see Fig 4B. For this choice of parameter values, the oscillation is illustrated in Fig 4C: period = 15.5, and the maximum value of *P*_tot_ = 540. Following a similar argument as that for the original model with *N* = 3, we write $3\times 10^{4}={V_{N}\hat{P}+V}_{C}({\hat{P}}_{1}+{\hat{P}}_{2}+\cdots +{\hat{P}}_{J})=V_{N}{\hat{K}}_{d}P+V_{C}{\hat{K}}_{d}(\frac{\hat{\beta }}{{\hat{\alpha }}_{J+2}}P_{J}+\ldots +\frac{{\hat{\beta }}^{J}}{{\hat{\alpha }}_{J+2}\ldots \alpha_{3}}P_{0})$. Assuming the identities ${\hat{\alpha }}_{3}=\ldots ={\hat{\alpha }}_{J+2}=\hat{\beta }$ (for a simple phosphorylation chain) and $\frac{\hat{\beta }}{{\hat{\alpha }}_{J+2}}=\frac{V_{N}}{V_{C}}$ (the conservation law for nuclear transport mentioned before, with ${\hat{\alpha }}_{3}$ replaced by ${\hat{\alpha }}_{J+2}$), we rewrite the relation above as $3\times 10^{4}=V_{N}{\hat{K}}_{d}(P+P_{J}+\ldots +P_{1})=V_{N}{\hat{K}}_{d}P_{tot}$. (When there is no chance of misunderstanding, we write *P*_tot_ for $\underset{t}{\max}\;P_{\mathrm{tot}}(t)$.) So, in this case we might estimate that ${\hat{K}}_{d}$ = 100 nM/540 ≈ 0.2 nM. However, ‘*P*_tot_’ includes *Per* mRNA species as well as PER protein species. So a better estimate of *P*_tot_ might be ‘300’, in which case ${\hat{K}}_{d}$ ≈ 0.33 nM, which is still 30-fold smaller than our estimate of ${\hat{K}}_{\mathrm{d,est}}$ = 10 nM for the binding of PER to BMAL1. Furthermore, in this case, we estimate ${\hat{A}}_{T}$ = 13 nM (4000 molecules in a nucleus of volume 500 fL), which is perhaps too small compared to the observed number of ~25,000 BMAL1 molecules.

#### Saturating degradation of nuclear PER

PER is degraded by proteasomes after it is poly-ubiquitinated by the E3 ligase β-TrCP [43]. Because the rate of this enzyme-mediated reaction likely saturates at large substrate concentration, it is reasonable to replace the linear kinetics for degradation of nuclear PER in Eq (3) by a Michaelis-Menten rate law [43],

$$\frac{dP}{dt}=P_{J}-\frac{\beta_{\max}P}{K_{m}+P}$$ (14’)

*β*_max_ and *K*_m_ are dimensionless parameters; in particular, $K_{m}={\hat{K}}_{m}/{\hat{K}}_{d},\beta_{\max}={\hat{\beta }}_{\max}/\left(\hat{\beta }{\hat{K}}_{d}\right)$. This change also has the potential to increase the oscillatory robustness of the model. Intuitively, the upper limit to the rate of PER degradation introduced by the Michaelis-Menten rate law causes nuclear PER concentration to react sluggishly to changes in the rate of *Per* mRNA production, which is another sort of ‘lag’ in the negative feedback loop.

To keep track of these changes, we introduce the notation SNF (0DN), where D denotes the PER degradation rate law (L for linear or M for Michaelian), and N denotes the number of species in the negative feedback loop. For example, the original KF model is denoted SNF (0L3). The significance of the ‘0’ will become evident shortly.

**Fig 5:**
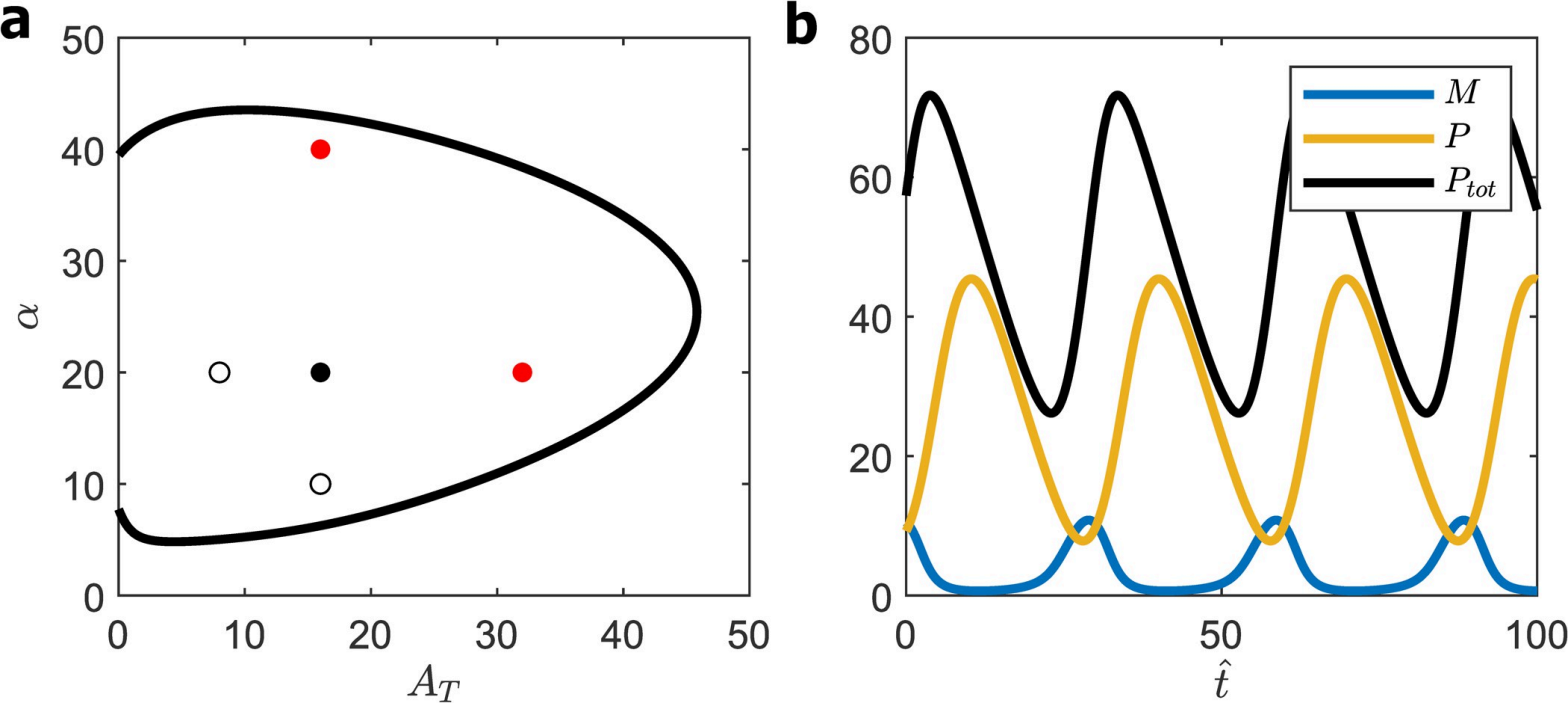
SNF (0M8) model. **(a)** Bifurcation diagram for *β*_max_ = 3.8, *K*_m_ = 1. Five-point criterion locates WT cell at the black dot. **(b)** Time-courses of *M*(*t*), *P*(t) and *P*_tot_(*t*) for WT cell: A_T_ = 16, *α* = 20; Period = 30, $\underset{t}{\max}\;P_{\mathrm{tot}}(t)$ = 70.

For the case of ‘saturating degradation,’ we still scale all concentrations with respect to ${\hat{K}}_{d}$, but we can no longer derive a closed-form algebraic equation for the locus of Hopf bifurcations. Instead, for *N* = 8, we searched the four-dimensional parameter space (*α*, *A*_T_, *β*_max_, *K*_m_) for oscillations with the smallest value of $\underset{t}{\max}\;P_{\mathrm{tot}}(t)$, subject to the constraints that *K*_m_ > 1 and that the model gives a reasonable domain of oscillations in the (*α*, *A*_T_) plane (i.e., large enough to satisfy the five-point criterion). We found (see S3 Text) several different combinations of *β*_max_ and *K*_m_ that could satisfy these criteria with similar values of $\underset{t}{\max}\;P_{\mathrm{tot}}(t)$, suggesting that the model is robust with respect to these criteria. A typical combination is *β*_max_ = 3.8 and *K*_m_ = 1, shown in Fig 5A. The five-point criterion is satisfied for $\left(\alpha^{\mathrm{WT}}=20,\;A_{T}^{\mathrm{WT}}=16\right)$, and the oscillations for this case are shown in Fig 5B, for which *P*_tot_ = 70; discounting for mRNA species, we estimate *P*_tot_ = 50. Hence, ${\hat{K}}_{d}$ = 100 nM/50 ≈ 2 nM and ${\hat{A}}_{T}$ ≈ 30 nM (9,000 molecules in a nucleus of volume 500 fL). This estimate of the theoretical value of ${\hat{K}}_{d}$ is now within our range of the probable experimental value, 1 nM < ${\hat{K}}_{d}$ < 10 nM, and the estimate of the total number of BMAL1 molecules per nucleus is acceptable, considering our uncertainty about the localization of BMAL1.

For further information, see S3 Text for notable patterns in the optimization results for SNF (0M8).

A disturbing property of this SNF (0M8) model is that oscillations persist even as *A*_T_ → 0, which is clearly impossible because there can be no expression of the *Per* gene when BMAL1 concentration is zero. The problem, of course, is that the rate law for *Per* transcription (rate ∝ *A*_free_/*A*_T_) is valid only if BMAL1 saturates *Per* E-boxes, which clearly cannot be true as *A*_T_ → 0. To get around this problem, we propose a revised rate law for *Per* gene transcription.

### A more realistic rate law for *Per* transcription does not affect the robustness of the SNF model

We propose to replace the KF expression for the rate of *Per* gene transcription (Rate Law 0) by a revised Rate Law 1 that is more realistic for small *A*_T_ (see S4 Text):

$$Rate\;Law\;0:\;\frac{dM}{dt}=\alpha \frac{A_{free}}{A_{T}}-M$$ (15-0)

$$Rate\;Law\;1:\;\frac{dM}{dt}=\alpha \frac{A_{\mathrm{free}}}{K_{A}+A_{T}}-M$$ (15-1)

For rate law 1, the maximum rate of transcription is $\alpha \frac{A_{T}}{K_{A}+A_{T}}$, which depends on how strongly BMAL1:CLOCK binds to the E-box, as characterized by the dimensionless dissociation constant $K_{A}={\hat{K}}_{A}/{\hat{K}}_{d}$; and also, when *A*_T_ becomes small, the transcription rate is proportional to *A*_free_/*K*_A_ (not *A*_free_/*A*_T_). Rate law 0 applies to the case in which binding between PER:CRY and BMAL1:CLOCK is independent of the binding between BMAL1:CLOCK and E-box, and BMAL1:CLOCK complexes saturate *Per* E-boxes. Rate law 1 relaxes the assumption of saturation of *Per* E-boxes by BMAL1:CLOCK.

#### Modified Kim-Forger SNF equations

Taking all of the aforementioned changes into account, we have (see S5 Text):

$$\frac{dM}{dt}=\alpha ∙F(A_{free})-M$$ (16)

$$\frac{dP_{1}}{dt}=M-P_{1}$$ (17)

$$\frac{dP_{j}}{dt}=P_{j-1}-P_{j},j=2,\ldots ,J$$ (18)

$$\frac{dP}{dt}=P_{J}-G(P)$$ (19)

$$A_{free}=\frac{1}{2}[A_{T}-P-1+\sqrt{{\left(A_{T}-P-1\right)}^{2}+4A_{T}}]$$ (20)

$$\mathrm{where}\;F(A_{\mathrm{free}})=\left\{ \begin{array}{c} A_{\mathrm{free}}/A_{T}\\ A_{\mathrm{free}}/{(K_{A}+A}_{T}) \end{array}\right.,\;\mathrm{and}\;G(P)=\left\{ \begin{array}{c} P\\ \beta_{\max}P/(K_{m}+P) \end{array}\right.$$ (21)

In the notation SNF(TDN), T denotes the *Per* transcription rate law (0 or 1), and *N* = *J* + 2 = total length of the negative feedback loop.

Models of form SNF (1LN) can be analyzed exactly as SNF(0LN), and the condition for a Hopf bifurcation is Eq (9), Φ∙*α*^2^−Ψ(*A*_T_)∙*α*+Ω(*A*_T_) = 0, where,

$$\Phi ={\left(S_{N}-1\right)}^{2},\Psi (A_{T})=S_{N}(\frac{A_{T}+K_{A}}{A_{T}})({\left(A_{T}+1\right)}^{2}-4S_{N}A_{T}),$$

$$\Omega (A_{T})={S_{N}}^{2}\frac{{\left(A_{T}+K_{A}\right)}^{2}}{A_{T}}{\left(A_{T}+1\right)}^{2}$$ (10”)

**Fig 6:**
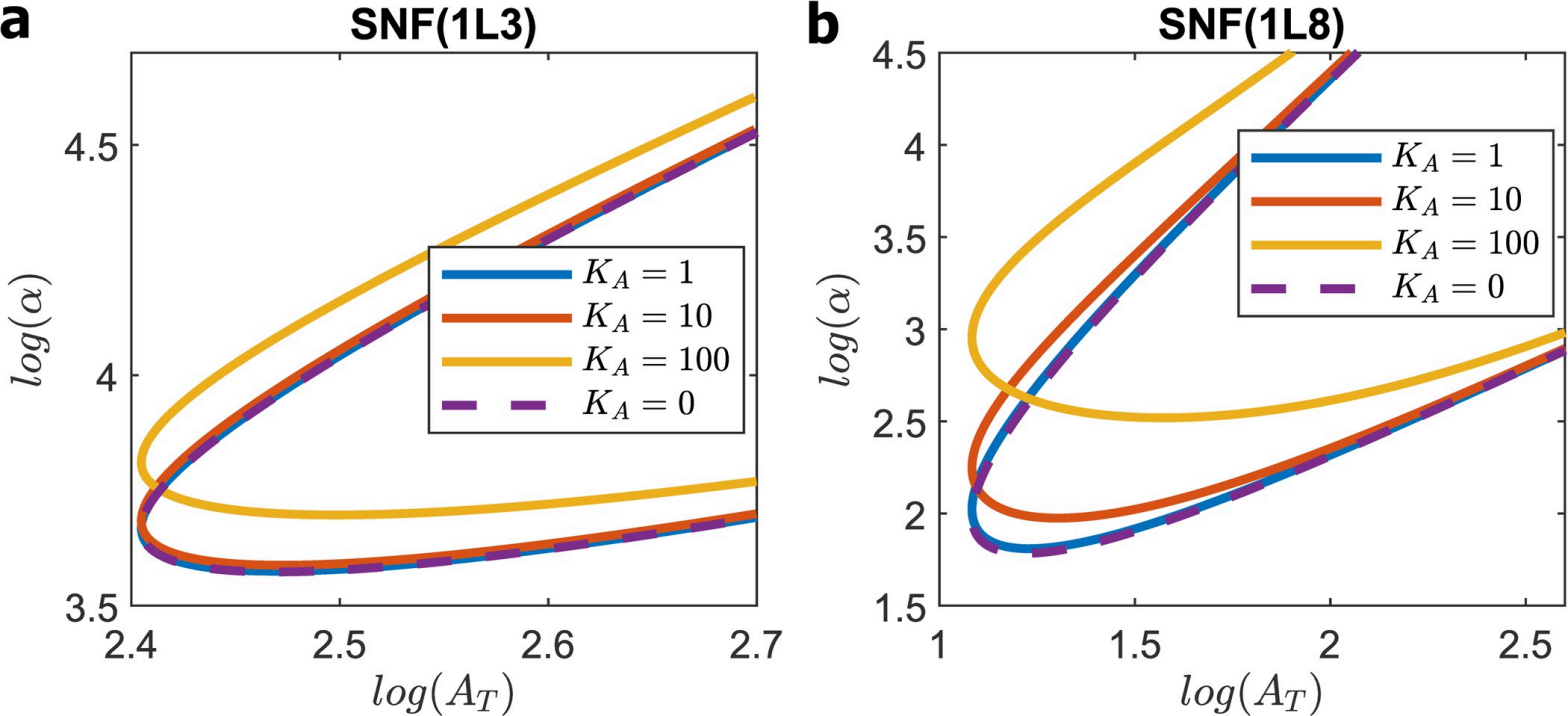
SNF (1LN) models. Loci of Hopf bifurcations for **(a)** SNF (1L3) and **(b)** SNF (1L8) models for *K*_A_ = 1, 10 and 100. For comparison, the dashed lines (for *K*_A_ = 0) show contours for SNF (0LN). Rate law 1 makes little change to the oscillatory domain until *K*_A_ > 10.

Solving this quadratic equation for *α* as a function of *A*_T_, we plot the locus of Hopf bifurcations for *N* = 3 and 8 in Fig 6A and 6B. Clearly, this change in rate law makes little difference in the robustness of oscillations for 1≤*K*_A_≤20. As *K*_A_ increases further, the bifurcation locus moves ‘up’ and *P*_tot_ increases, so the estimated value of ${\hat{K}}_{d}$ gets smaller.

**Fig 7:**
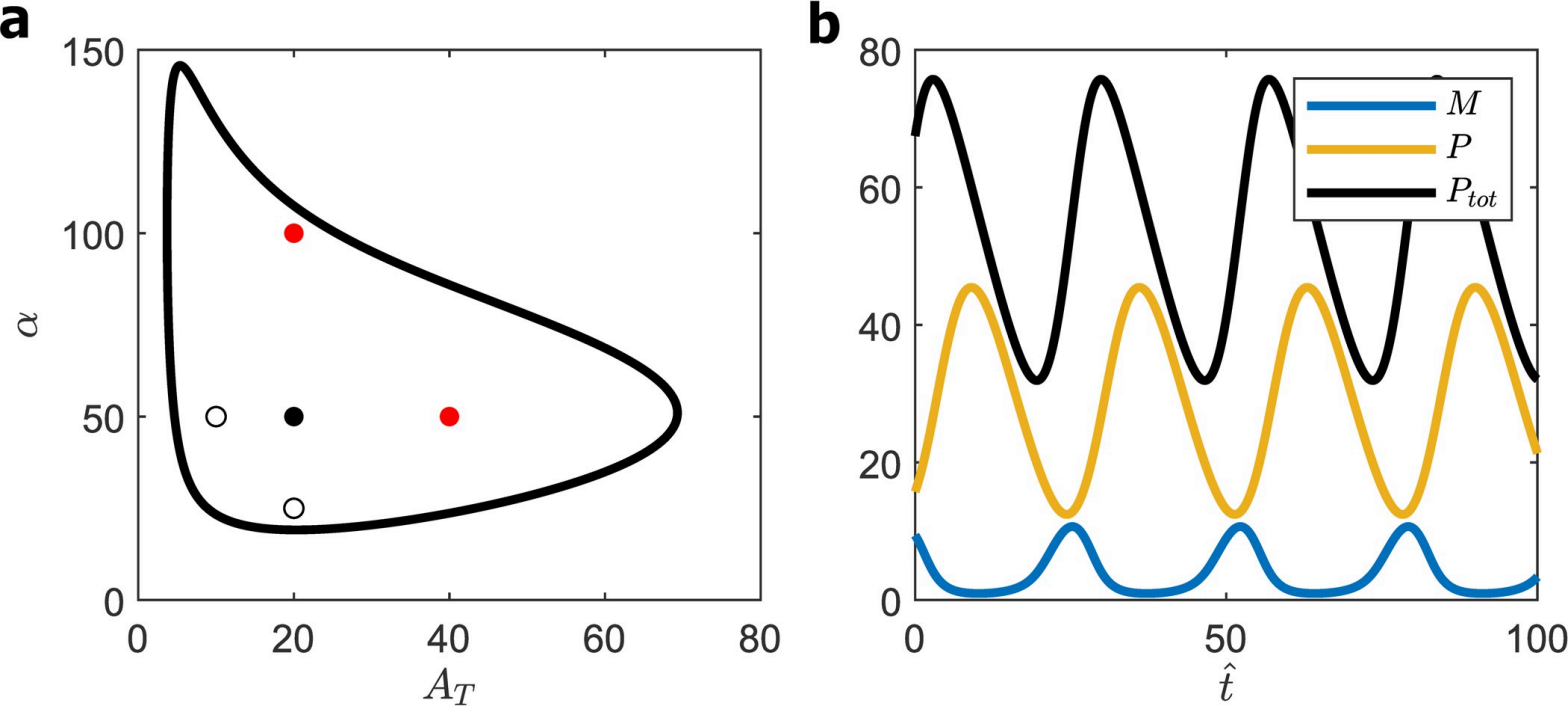
SNF (1M8) model. **(a)** Bifurcation diagram for *β*_max_ = 5, *K*_m_ = 5.5, *K*_A_ = 20. Five-point criterion locates WT cell at the black dot. **(b)** Time-courses of *M* (*t*), *P*(t) and *P*_tot_(*t*) for WT cell: A_T_ = 20, *α* = 50; Period = 27, $\underset{t}{\max}\;P_{\mathrm{tot}}(t)$ = 75.

For SNF (1MN) we have no closed-form algebraic equation for the locus of Hopf bifurcations, so as before, we set *N* = 8 and searched the five-dimensional parameter space (*α*, *A*_T_, *β*_max_, *K*_m_, *K*_A_) for oscillations with the smallest value of $\underset{t}{\max}\;P_{\mathrm{tot}}(t)$, subject to the constraints

$$\alpha \in [10^{-2},10^{3}],A_{T}\in [10^{-2},10^{2}],\beta_{\max}\in [10^{-2},10^{3}],K_{m}\in [1,10^{2}],K_{A}\in [1,10^{2}],$$

and that the amplitude of oscillation of *P*_tot_(*t*) be larger than 0.5, where $ampl=\frac{max-min}{max+min}$. The amplitude constraint is to select for ‘robust’ oscillations. A summary of these calculations is provided in S3 Text. Briefly, we found ~1000 parameter sets with $\underset{t}{\max}\;P_{\mathrm{tot}}(t)$ = 32.3 ± 3.5 and Period = 20.7 ± 1.1. Then we checked for parameter sets that satisfy the ‘five-point criterion’. Results of a typical parameter set (*β*_max_ = 5, *K*_m_ = 5.5 and *K*_A_ = 20) are illustrated in Fig 7A and 7B. In this case, $\underset{t}{\max}\;P_{\mathrm{tot}}(t)$ = 75, which is larger than ‘33’ because the WT cell $\left(\alpha^{\mathrm{WT}}=50,A_{T}^{\mathrm{WT}}=20\right)$ must be centered in the oscillatory domain of Fig 7A. Discounting *P*_tot_ for mRNA species, we estimate $\underset{t}{\max}\;P_{\mathrm{tot}}(t)$ ≈ 40. Hence, ${\hat{K}}_{d}$ = 2.5 nM and ${\hat{A}}_{T}$ = 50 nM (~15,000 molecules of BMAL1 per nucleus). We conclude that, although rate law 1 is more accurate than rate law 0 for values of *A*_T_ ≤ *K*_A_, it does not improve significantly on our estimates of ${\hat{K}}_{d}$ and ${\hat{A}}_{T}$.

### Adding a negative feedback loop involving REV-ERB does not increase the robustness of circadian oscillations

Next, we explore Kim & Forger’s NNF model (see S2 Text), with modified rate laws for gene transcription. For the rates of transcription of *Per* and *Rev-erb* genes governed by BMAL1:CLOCK binding to E-boxes, we use our Rate Law 1, Eq (15-1). For the transcriptional repression of the *Bmal1* gene by REV-ERB (variable $\hat{V}$), we replace Kim & Forger’s function *γ/V* by ${\hat{A}}_{\mathrm{MAX}}∙{\hat{K}}_{V}/(\hat{V}+{\hat{K}}_{V})$, where ${\hat{K}}_{V}$ is the dissociation constant for REV-ERB binding to the promoter (RORE) of the *Bmal1* gene. This new rate law remedies an issue in KF’s original NNF model, for which the rate of synthesis of *Bmal1* mRNA → ∞ as *V*→0.

#### Modified Kim-Forger NNF model

Eqs (16)–(21) plus

$$\frac{d{\hat{A}}_{T}}{d\hat{t}}=\hat{\delta }[{\hat{A}}_{MAX}\frac{{\hat{K}}_{V}}{\hat{V}+{\hat{K}}_{V}}-{\hat{A}}_{T}]\;\frac{dA_{T}}{dt}=\delta [A_{MAX}\frac{1}{V+1}-A_{T}]$$ (22)

$$\frac{d\hat{V}}{d\hat{t}}=\hat{\delta }[{\hat{V}}_{MAX}F({\hat{A}}_{\mathrm{free}})-\hat{V}]\;\frac{dV}{dt}=\delta [V_{MAX}F(A_{\mathrm{free}})-V]$$ (23)

The dimensional equations on the left-hand-side are cast into dimensionless form with the same definitions used in Eqs (1)–(4), plus $\delta =\frac{\hat{\delta }}{\hat{\beta }}$, $A_{\mathrm{MAX}}=\frac{{\hat{A}}_{\mathrm{MAX}}}{{\hat{K}}_{d}},V=\frac{\hat{V}}{{\hat{K}}_{V}},V_{\mathrm{MAX}}=\frac{{\hat{V}}_{\mathrm{MAX}}}{{\hat{K}}_{V}}.$

To compare NNF to SNF, we must adopt some constraints on the new parameters. First of all, from Narumi et al. [36], we find that the maximum number of REV-ERB molecules during the circadian rhythm in mouse liver cells is 50,000. If all molecules are confined to a nucleus of 500 fL, then $\frac{50,000\;\mathrm{molec}}{500\;\mathrm{fL}}=167\;\mathrm{nM}={\hat{K}}_{V}∙\underset{t}{\max}\;V(t)$. For ${\hat{K}}_{V}$ to be greater than, say, 10 nM, we will constrain *V*_MAX_ so that $\underset{t}{\max}\;V(t)<10$. We continue to insist that the relative amplitude of *P*_tot_(*t*) be > 0.5, and, in addition, we constrain *A*_MAX_ so that the relative amplitude of *A*(*t*) over an oscillation is > 0.2 [36]. We also require that $\underset{t}{\max}\;A_{T}(t)/\underset{t}{\max}\;P_{\mathrm{tot}}(t)$ be as close to 1 as possible (total numbers of BMAL1 and PER proteins are close [36]). Subject to these constraints, we search over the available parameter space

$$\alpha \in [10^{-2},10^{3}],A_{\mathrm{MAX}}\in [10^{-2},10^{3}],\beta_{\max}\in [10^{-2},10^{3}],K_{m}\in [1,10^{2}],K_{A}\in [1,10^{2}],$$

$$V_{\max}\in [10^{-2},10^{2}],\delta \in [10^{-2},10^{2}]$$

to minimize the objective function $\underset{t}{\max}\;P_{\mathrm{tot}}(t)$; i.e., to maximize the value of ${\hat{K}}_{d}$.

**Fig 8:**
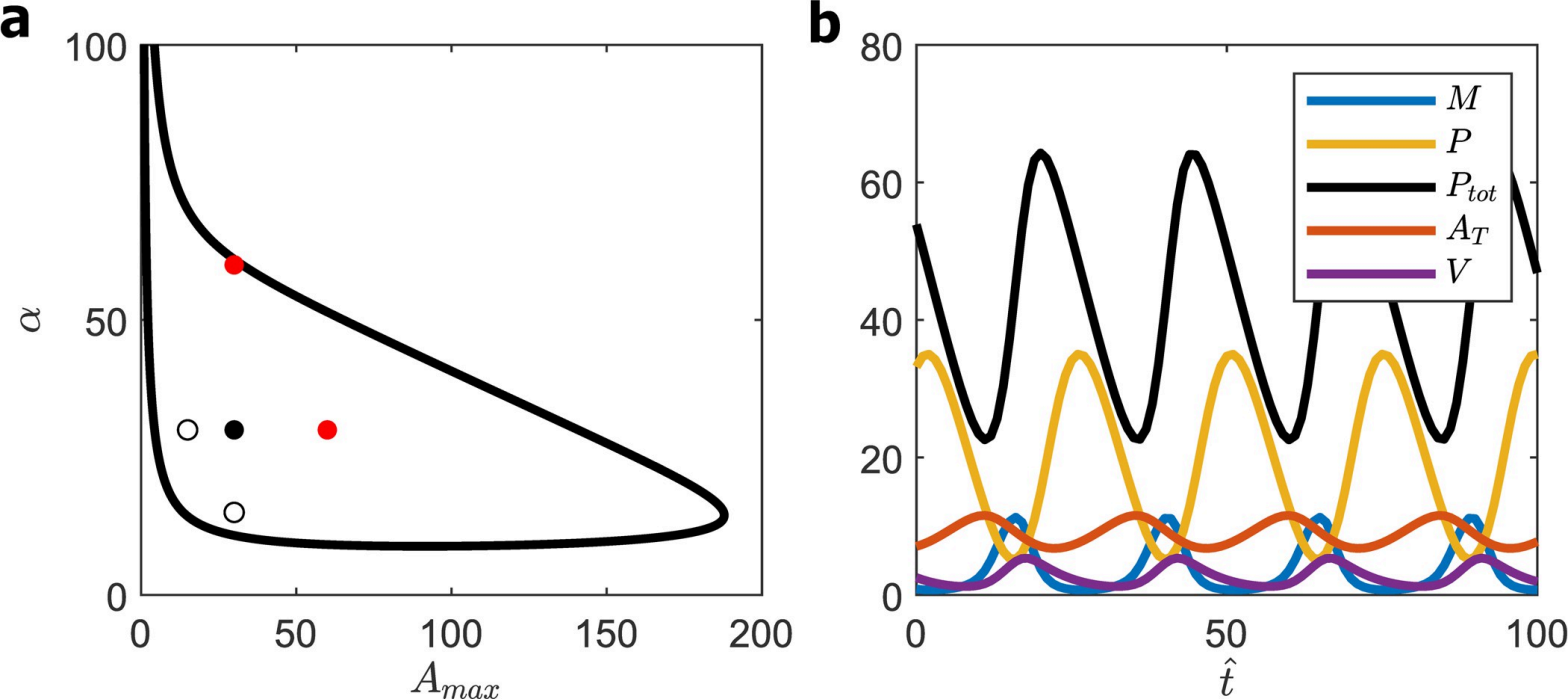
NNF (1M8) model. **(a)** Bifurcation diagram for *β*_max_ = 4.5, *K*_m_ = 2.5, *K*_A_ = 3.7, *V*_MAX_ = 22, *δ* = 0.17. Five-point criterion locates WT cell at the black dot. **(b)** Time-courses of *A*_T_(*t*), *V*(*t*), *M*(*t*), *P*(t) and *P*_tot_(*t*) for WT cell: *A*_MAX_ = 30, *α* = 30; Period = 25, $\underset{t}{\max}\;P_{\mathrm{tot}}(t)$ = 64, avg(*A*_T_) = 9.

A summary of these calculations is provided in S3 Text. Briefly, we found ~1000 parameter sets with $\underset{t}{\max}\;P_{\mathrm{tot}}(t)$ ≈ 91.7 ± 24.5 and Period = 22.7 ± 2.1. Again, after checking for parameter sets that satisfy the ‘five-point criterion’, we plot results of a typical parameter set (*β*_max_ = 4.5, *K*_m_ = 2.5, *K*_A_ = 3.7, *V*_MAX_ = 22 and *δ* = 0.17) in Fig 8. For the WT cell $(\alpha^{\mathrm{WT}}=30,A_{\mathrm{MAX}}^{\mathrm{WT}}=30),\underset{t}{\max}\;P_{\mathrm{tot}}(t)$ = 64 and avg(*A*_T_) = 9. Discounting for mRNA species, $\underset{t}{\max}\;P_{\mathrm{tot}}(t)$ = 35, ${\hat{K}}_{d}$ = 3 nM and ${\mathrm{avg}(\hat{A}}_{T})$ = 27 nM (~8,000 molecules of BMAL1 per nucleus). We conclude that NNF (1M8) is not more robust than SNF(1M8), nor does it improve our estimates of ${\hat{K}}_{d}$ and ${\hat{A}}_{T}$.

### An additional positive feedback loop involving ROR increases the robustness of circadian oscillations at a cost

Next, we explore Kim & Forger’s PNF model, with similarly modified rate laws for gene transcription: Eq (15-1) for the rates of transcription of *Per* and *Ror* genes, and ${\hat{A}}_{\mathrm{MAX}}∙(\varepsilon {\hat{K}}_{R}+\hat{R})/(\hat{R}+{\hat{K}}_{R})$ for the rate of transcription of *Bmal1* by ROR (variable $\hat{R}$). In the latter rate law, ${\hat{K}}_{R}$ is the dissociation constant for ROR binding to the RORE promoter and *ε* is the fractional reduction in *Bmal1* expression when $\hat{R}=0$. This new rate law remedies an issue in KF’s original PNF model, for which the rate of *Bmal1* transcription does not behave appropriately as *R*→∞ or *R*→0.

#### Modified Kim-Forger PNF model

Eqs (16)–(20) plus

$$\frac{d{\hat{A}}_{T}}{d\hat{t}}=\hat{\delta }[{\hat{A}}_{MAX}\frac{\varepsilon {\hat{K}}_{R}+\hat{R}}{\hat{R}+{\hat{K}}_{R}}-{\hat{A}}_{T}]\;\frac{dA_{T}}{dt}=\delta [A_{MAX}\frac{\varepsilon +R}{R+1}-A_{T}]$$ (24)

$$\frac{d\hat{R}}{d\hat{t}}=\hat{\delta }[{\hat{R}}_{MAX}F({\hat{A}}_{\mathrm{free}})-\hat{R}]\;\frac{dR}{dt}=\delta [R_{MAX}F(A_{\mathrm{free}})-R]$$ (25)

The dimensional equations on the left-hand-side are cast into dimensionless form with the same definitions used earlier, plus $R=\frac{\hat{R}}{{\hat{K}}_{R}},R_{\mathrm{MAX}}=\frac{{\hat{R}}_{\mathrm{MAX}}}{{\hat{K}}_{R}}.$

To compare PNF to SNF, we adopt the following constraints. First of all, since the maximum number of ROR molecules during the circadian rhythm in mouse liver cells is 25,000 [36], we estimate that $\frac{25,000\;\mathrm{molec}}{500\;\mathrm{fL}}=83\;\mathrm{nM}={\hat{K}}_{R}∙\underset{t}{\max}\;R(t)$, and consequently we constrain *R*_MAX_ so that $\underset{t}{\max}\;R(t)<5$. We continue to insist that the relative amplitudes of *P*_tot_(*t*) be > 0.5 and of *A*(*t*) be > 0.2, and that $\underset{t}{\max}\;A_{T}(t)/\underset{t}{\max}\;P_{\mathrm{tot}}(t)$ be as close to 1 as possible. Subject to these constraints, we search over the available parameter space

$$\alpha \in [10^{-2},10^{3}],A_{\mathrm{MAX}}\in [10^{-2},10^{3}],\beta_{\max}\in [10^{-2},10^{3}],K_{m}\in [1,10^{2}],K_{A}\in [1,10^{2}],$$

$$R_{\max}\in [10^{-2},10^{2}],\delta \in [10^{-2},10^{2}],\varepsilon \in [10^{-4},10^{-1}]$$

to minimize the objective function $\underset{t}{\max}\;P_{\mathrm{tot}}(t)$.

**Fig 9:**
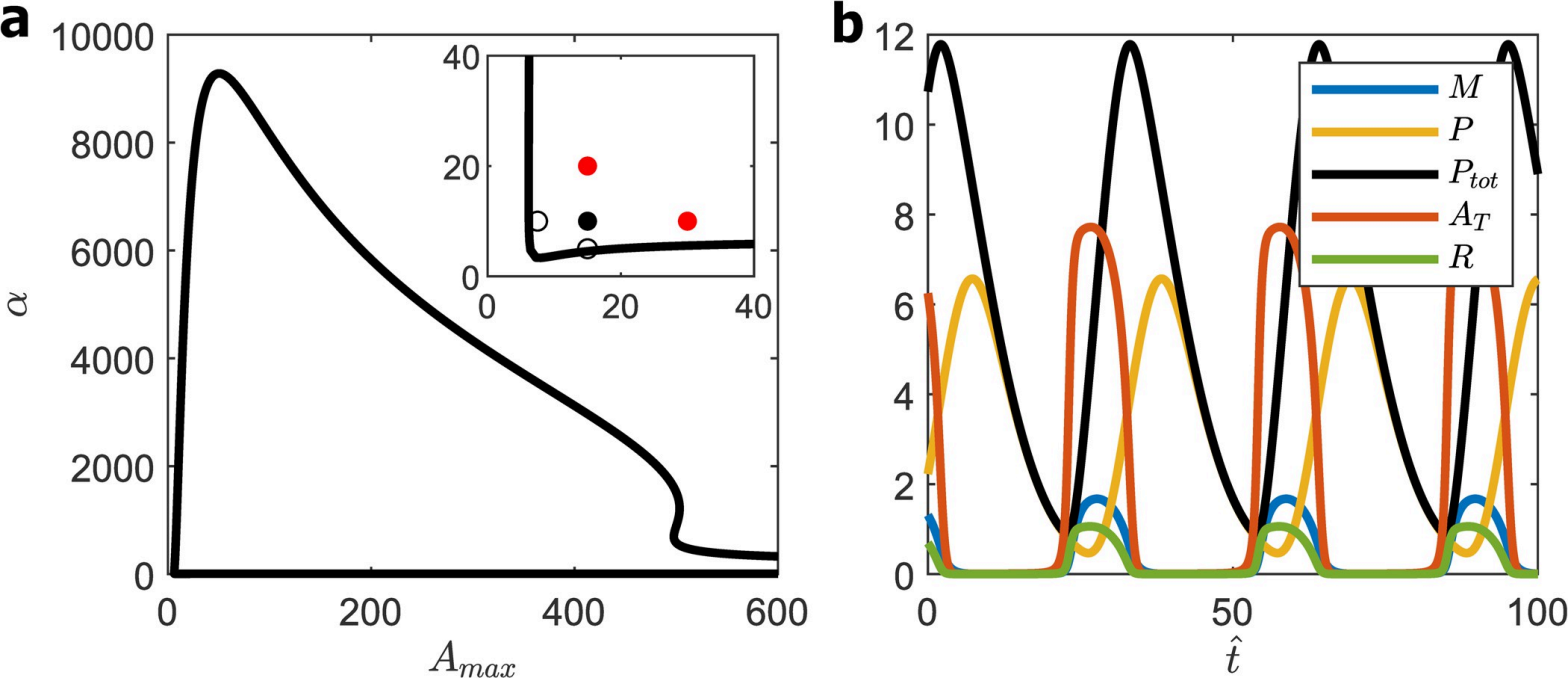
PNF (1M8) model. **(a)** Bifurcation diagram for *β*_max_ = 1.85, *K*_m_ = 8.5, *K*_A_ = 35, *R*_MAX_ = 6.2, *ε* = 0.0003, *δ* = 8. Five-point criterion locates WT cell at the black dot. **(b)** Time-courses of *M* (*t*), *P*(t), *A*_T_(*t*), *R*(*t*), and *P*_tot_(*t*) for WT cell: *A*_MAX_ = 15, *α* = 10. Period = 31, $\underset{t}{\max}\;P_{\mathrm{tot}}(t)$ = 12, avg (*A*_T_) = 3.

A summary of these calculations is provided in the S3 Text. Briefly, we found ~1000 parameter sets with $\underset{t}{\max}\;P_{\mathrm{tot}}(t)$ ≈ 3.6 ± 2.2 and Period = 43.9 ± 25.4. Again, after checking for parameter sets that satisfy the ‘five-point criterion’, we plot results of a typical parameter set (*β*_max_ = 1.85, *K*_m_ = 8.5, *K*_A_ = 35, *R*_MAX_ = 6.2, *ε* = 0.0003 and *δ* = 8) in Fig 9. For the WT cell $(\alpha^{\mathrm{WT}}=10,A_{\mathrm{MAX}}^{\mathrm{WT}}=15),\underset{t}{\max}\;P_{\mathrm{tot}}(t)$ = 12 and ${\mathrm{avg}(\hat{A}}_{T})$ = 3. Discounting for mRNA species, $\underset{t}{\max}\;P_{\mathrm{tot}}(t)$ = 7; hence, ${\hat{K}}_{d}$ = 15 nM and ${avg(\hat{A}}_{T})$ ≈ 45 nM (i.e., 13,000 molecules of BMAL1 per nucleus), which are quite reasonable estimates. Clearly, the PNF (1M8) exhibits more robust oscillations than the SNF(1M8) and NNF(1M8) models and is consistent with ${\hat{K}}_{d}$ ≈ 10 nM, but the oscillation waveforms are unbelievable. For about half of the oscillatory period, *R*(*t*), *A*_T_(*t*) and *M*(*t*) are ≈ 0, which is inconsistent with observations [36].

### The three 1M8 models are about equally robust with respect to circadian oscillations

**Fig 10:**
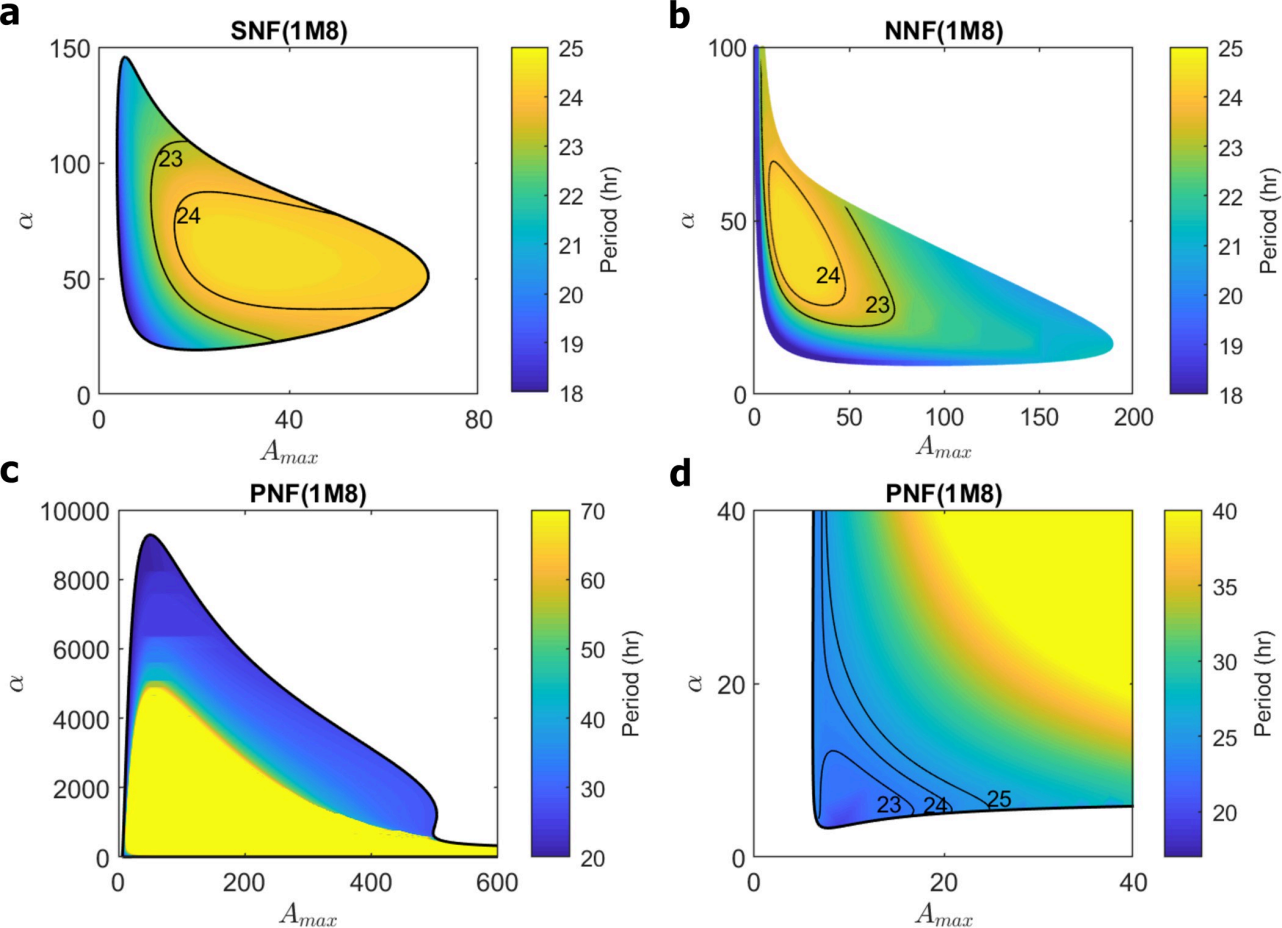
Distributions of oscillatory period for 1M8 models. **(a)** SNF (1M8) for *β*_max_ = 5, *K*_m_ = 5.5, *K*_A_ = 20; WT cell at A_T_ = 20, *α* = 50, ${\hat{\beta }}_{1}=1.125\;h^{-1}$, so that WT cell exhibits 24 h rhythm. **(b)** NNF (1M8) for *β*_max_ = 4.5, *K*_m_ = 2.5, *K*_A_ = 3.7, *V*_MAX_ = 22, *δ* = 0.17; WT cell at A_T_ = 30, *α* = 30, ${\hat{\beta }}_{1}=1\;h^{-1}$, so that WT cell exhibits 25 h rhythm. **(c)** PNF (1M8) for *β*_max_ = 1.85, *K*_m_ = 8.5, *K*_A_ = 35, *R*_MAX_ = 6.2, *ε* = 0.0003, *δ* = 8; WT cell at A_MAX_ = 15, *α* = 10, ${\hat{\beta }}_{1}=1.3\;h^{-1}$, so that WT cell exhibits 24 h rhythm. **(d)** Lower left corner of panel c.

In Fig 10 we redraw the bifurcation plots for the ‘1M8’ models with colors to indicate oscillatory periods. (For each model, we choose a value of ${\hat{\beta }}_{1}$, as indicated in the legend, to convert from dimensionless period *τ* to a period of ~24 h given the wild-type parameter values, specified in the legend.) For SNF and NNF models the oscillatory period varies over a range of ~22–25 h. The PNF model, though very robust in terms of oscillatory potential, is restricted in exhibiting circadian oscillations (say, 23–26 h) to two regions of expression of BMAL1 (parameter *A*_MAX_) and PER (parameter *α*); namely, a broad band around the diagonal *α* + 16∙*A*_MAX_ ≈ 8000, which is clearly seen in Fig 10C, and a triangular region *α* + *A*_MAX_ < 35 (for 5 < *A*_MAX_ < 35, 5 < *α* < 35) seen in Fig 10D. Our WT simulation (Fig 9) is found in the ‘triangular region.’

Oscillations in the ‘broad band’ (results not shown), although they are consistent with 1 nM < ${\hat{K}}_{d}$ < 10 nM, predict average concentrations of nuclear BMAL1 that are much too small (< 1 nM = 300 molecules per nucleus). Indeed, $\underset{t}{\max}\;A_{T}(t)$ is so small despite *A*_MAX_ being very large, because the concentration of ROR in this region is very small ($\underset{t}{\max}\;R(t)<0.003$) and *dA*_T_/*dt*≈*A*_MAX_(*ε*+*R*)<0.003∙*A*_MAX_. We note, in passing, that robust oscillations and a broad distribution of periods, as we see in the PNF model, is a common feature of models that combine positive and negative feedback loops [42,44].

We can quantify the ‘robustness’ of the 1M8 models by measuring the area of ‘circadian oscillations’ in the (*A*_T_, *α*) or (*A*_MAX_, *α*) plane. To standardize the area, we measure *A*_T_ (*A*_MAX_) and *α* as multiples of their WT values, as given in the figure legend. In these units, the areas are NNF:SNF:PNF = 6.6:4:2. These ratios are probably not significantly different in terms of what might possibly be measured experimentally.

In S2 Fig we explore the dependence of oscillatory period on fold-changes in BMAL1 expression (i.e., co-expression of BMAL1 and CLOCK in experiments). SNF (0LN) models are quite insensitive to fold-changes in BMAL1 expression: the change in period is ~1 h across the range of oscillations. SNF (0M8), SNF (1M8) and NNF (1M8) models are more sensitive, with a change of 5–8 h across the range. Apparently, the saturating rate law for nuclear PER degradation is responsible for the increased sensitivity to variable expression of BMAL1. PNF (1M8) exhibits very long periods of oscillation for large values of *A*_MAX_, as noted, and is comparably sensitive (ΔT ≈ 8 h) over a restricted range of BMAL1 overexpression (up to 2.5 x WT level). When Lee et al. constitutively overexpressed BMAL1:CLOCK dimers (~ four-fold) in MEF cells, they observed rhythms of reduced amplitude but normal 24 h period (see their Fig 3C) [34]. This observation is within the limits predicted by models with linear degradation of nuclear PER but is not consistent with the assumption of saturating degradation.

S3 Fig shows the trends with respect to PER overexpression (e.g., co-expression of PER2 and CRY1 in experiments). SNF (0L8) is quite insensitive (ΔT ≈ 1 h), whereas SNF (0M8) and SNF(1M8) are more sensitive (ΔT ≈ 4.5 h). NNF (1M8) and PNF (1M8) are even more sensitive to overexpression of PER (8–10 h). These trends are subject to experimental investigation by overexpression of BMAL1:CLOCK and PER2:CRY1.

## Discussion

The Kim-Forger (KF) models of mammalian circadian rhythms (called SNF, NNF and PNF) are appealing in many respects, but they rely on an unrealistic requirement for robust oscillations, namely that the equilibrium dissociation constant of the PER:CRY::BMAL1:CLOCK complex must be ${\hat{K}}_{d}$ < 0.04 nM, which is 250-fold smaller than a reasonable value for the dissociation of the PER:CRY::BMAL1:CLOCK complex. This difficulty can be ameliorated by lengthening the core negative feedback loop between *Per* mRNA transcription and PER:CRY inactivation of BMAL1:CLOCK (the transcription factor driving *Per* expression), and/or by implementing a Michaelis-Menten rate law for the degradation of nuclear PER. The KF models were further modified by introducing an alternative rate law for BMAL1:CLOCK-mediated transcription of clock genes (*Per*, *Rev-erb* and *Ror*) to correct a problem at low expression of the *Bmal1* gene, and by providing more accurate rate laws for the effects of REV-ERB and ROR on *Bmal1* expression.

With these modifications, we find (Fig 7) that the SNF (1M8) model can exhibit oscillations for ${\hat{K}}_{d}\approx 2\;\mathrm{nM}$. From biophysical considerations and *in vitro* measurements, we estimate that ${\hat{K}}_{\mathrm{d,est}}\approx 10\;\mathrm{nM}$, so the model constraint is not too far off from expectations. For ${\hat{K}}_{d}=2\;\mathrm{nM}$, the SNF (1M8) model oscillates over a 14-fold range of total BMAL1 concentrations, 10 nM < ${\hat{A}}_{T}$ < 140 nM. For ${\hat{A}}_{T}$ = 30 nM, the corresponding number of BMAL1 molecules in a nucleus of volume 500 fL would be $(30\;nM\times 500\;fL)(\frac{6\times 10^{14}}{1\;nmol})(\frac{10^{-15}L}{1\;fL})=9,000$, which is about one-third the observed number (~25,000) of BMAL1 molecules in a mammalian cell [36]. If the remaining BMAL1 molecules are dispersed through the cytoplasm of volume 5000 fL, the cytoplasmic concentration of BMAL1 would be about one-tenth the nuclear concentration, which is not unreasonable for a ‘nuclear’ protein such as BMAL1. Furthermore, the model focuses on BMAL1:CLOCK complexes that bind E-boxes to regulate gene expression. BMAL1 in this form may account for only a fraction of total BMAL1, if BMAL1, like PER, undergoes multi-step post-translational modifications. Indeed, both BMAL1 and CLOCK are known to be phosphorylated at multiple sites, which affects their stability and nuclear accumulation, as well as activity of the BMAL1:CLOCK complex [45–47].

Replacing the linear rate law for nuclear PER degradation by a Michaelis-Menten rate law causes a dramatic change in the sensitivity of oscillation to the expression levels of *Per2* and *Bmal1* (compare Figs 4A and 5A). Models with linear PER degradation, e.g., SNF (0L8), predict that oscillations are possible over an ever broadening range of rates of *Per* and *Bmal1* expression; e.g., $1.8<\frac{\alpha -50}{A_{T}-10}<75$ (approximately) in Fig 4A. For a comparable model with Michaelis-Menten degradation, SNF (0M8), the oscillatory domain is bounded by maximal rates of expression: *α* < 45 and *A*_T_ < 45 in Fig 5A. These contrasting results provide a testable prediction for future experimental exploration. By overexpressing *Per/Cry* genes and/or *Bmal1/Clock* genes under control of their normal (regulated) promoters (i.e., by manipulating *α* and *A*_T_), one could test whether nuclear PER degradation operates in a saturated (Michaelis-Menten) or unsaturated (linear) manner, which would be difficult to measure directly *in vivo*. In the same experiment, by measuring the dependence of oscillation period on *α* and *A*_T_ (i.e., on fold-changes in expression of *Per*/*Cry* and *Bmal1*/*Clock*), one could investigate a second property of our models (S2 and S3 Figs) that period length is much more sensitive to *α* and to *A*_T_ in models with saturated degradation than in models with linear degradation of nuclear PER.

The single negative feedback loop (SNF), whereby PER inhibits its own synthesis, can be supplemented with an auxiliary positive feedback from ROR (PNF) or a second negative feedback from REV-ERB (NNF) on the synthesis of BMAL1. For their versions of these three models (0L3 versions), Kim & Forger observed a ‘robustness trend’ NNF > SNF > PNF, in terms of the size of the oscillatory domain in parameter space. For our versions of these models, we find that SNF and NNF have similar oscillatory domains, while PNF is much more robust. However, if ‘robustness’ is defined as the size of the domain of circadian oscillations (22–26 h) in parameter space (fold-changes in expression of PER:CRY and BMAL1:CLOCK complexes), then the SNF(1M8), NNF(1M8) and PNF(1M8) models are nearly equally robust.

Our models could be employed in the future to explore other features of the mammalian circadian clock. For instance, following the lead of Kim and colleagues [48,49], we could address our models to the circadian clock’s temperature-compensation and/or phase-shifting properties. Adding these key features may answer some remaining questions about the behaviors of these models. Another question that could be addressed with these models is the function of an anti-sense transcript of the PER2 gene [50]. Furthermore, these models could be applied in chronopharmacology and chronotherapy studies [51]. One such application would be modeling PER2’s interaction with the tumor suppressor protein p53 in stressed (e.g., DNA damage) cells compared to unstressed cells [52,53].

## Materials and methods

The ‘wiring diagrams’ (molecular mechanisms) of our models (see Fig 2) were converted into nonlinear ODEs, as described in the main text. The ODEs were solved by standard numerical algorithms, as implemented in MATLAB and XPP-AUTO. Software codes are provided in S1 and S2 Codes for XPP and MATLAB, respectively. Bifurcation diagrams were computed using XPP-Auto, which may be downloaded from www.math.pitt.edu/~bard/xpp/xpp.html. To optimize the parameters of SNF (0M8), SNF (1M8), NNF (1M8) and PNF (1M8) models, we used MATLAB’s simulated annealing method (‘simulannealbnd’) within physiologically reasonable ranges. The parameter ranges and optimization criteria we used for each model and the corresponding cost functions are provided in S3 Text.

## Supporting information

S1 FigBulk average of asymmetric oscillatory trajectories appears sinusoidal.Thin colored lines: *M*(*t*) trajectory in Fig 3 with a random shift in phase. The random phase was drawn from a normal distribution with zero mean and standard deviation of 0.5 time unit (~ 1/10 of the oscillation period). Thick black line: average of the colored trajectories. Skewness of a single colored trajectory and the average trajectory is 0.42 and 0.17, respectively. Skewness is defined as $S=\langle {\left(M-\langle M\rangle \right)}^{3}\rangle /{\langle {\left(M-\langle M\rangle \right)}^{2}\rangle }^{\frac{3}{2}}$.(DOCX)

S2 FigDependence of oscillation period on level of expression of *Bmal1*, either *A*_T_ for SNF models or *A*_MAX_ for NNF and PNF models.In the insets we record a measure of the relative change in period, $\Delta =\frac{T_{\max}-T_{\min}}{(T_{\max}+T_{\min})/2}$, across the range of gene expression, and the absolute change (in hours): Δ*T* = Δ∙24 h. For PNF(1M8) we limit the increase in gene expression to 2.5 x WT value of *A*_MAX_.(DOCX)

S3 Fig Dependence of oscillation period on level of expression of *Per*, i.e., parameter *α*. Δ and Δ*T*, the relative and absolute changes in period over the range of gene expression, are defined in the legend to S2 Fig. For PNF(1M8) we limit the increase in gene expression to 2.5 x WT value of *α*.
(DOCX)

S1 Table Definitions of the dynamical variables in the models. (DOCX)

S2 Table Definitions of the dimensionless parameters in the models. (DOCX)

S1 Text Goodwin’s model. (DOCX)

S2 Text Kim & Forger’s extended models. (DOCX)

S3 Text Parameter optimization for models with saturating degradation of nuclear PER. (DOCX)

S4 Text Deriving the rate laws for Per transcription. (DOCX)

S5 Text Non-dimensionalization of the modified Kim-Forger equations. (DOCX)

S1 Code Ode files for simulation and bifurcation analysis of every model using XPP-AUTO. (DOCX)

S2 Code Zip file of MATLAB codes for opimization of ‘M-type’ models. (ZIP)
