# Supplementary material for: Mathematical analysis of robustness of oscillations in models of the mammalian circadian clock
Source: PLoS Comput Biol. 2022 Mar 18;18(3):e1008340. doi: 10.1371/journal.pcbi.1008340 (PMC8979472; doi:10.1371/journal.pcbi.1008340)
Supplement: S1 Fig — Thin colored lines: M(t) trajectory in Fig 3 with a random shift in phase. The random phase was drawn from a normal distribution with zero mean and standard deviation of 0.5 time unit (~ 1/10 of the oscillation period). Thick black line: average of the colored trajectories. Skewness of a single colored trajectory and the average trajectory is 0.42 and 0.17, respectively. Skewness is defined as S=〈(M−〈M〉)3〉/〈(M−〈M〉)2〉32. (DOCX) [file pcbi.1008340.s001.docx]

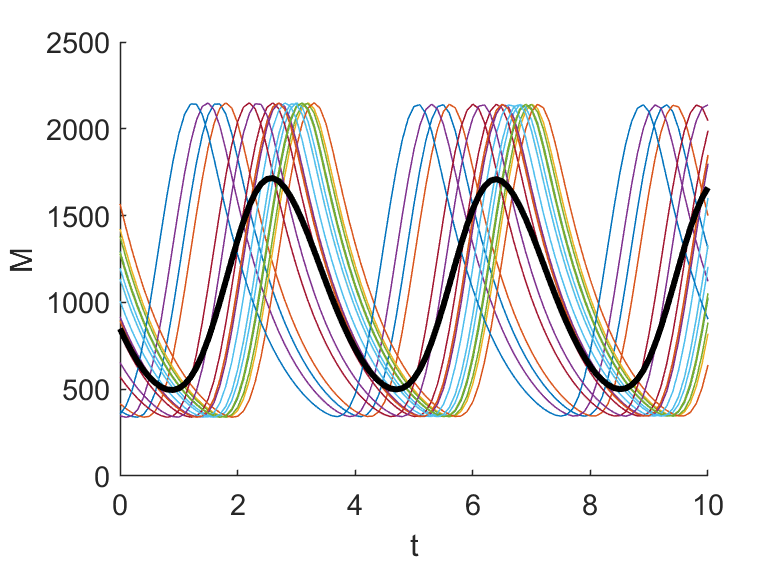


**S1 Fig.** **Bulk average of asymmetric oscillatory trajectories appears sinusoidal**. Thin colored lines: *M*(*t*) trajectory in Fig 3 with a random shift in phase. The random phase was drawn from a normal distribution with zero mean and standard deviation of 0.5 time unit (~ 1/10 of the oscillation period). Thick black line: average of the colored trajectories. Skewness of a single colored trajectory and the average trajectory is 0.42 and 0.17, respectively. Skewness is defined as $S=\left\langle\left( M-\left\langle M \right\rangle\right)^{3} \right\rangle/{\left\langle\left( M-\left\langle M \right\rangle\right)^{2} \right\rangle^{\frac{3}{2}}}$.
