# Supplementary material for: Mathematical analysis of robustness of oscillations in models of the mammalian circadian clock
Source: PLoS Comput Biol. 2022 Mar 18;18(3):e1008340. doi: 10.1371/journal.pcbi.1008340 (PMC8979472; doi:10.1371/journal.pcbi.1008340)
Supplement: S2 Fig — In the insets we record a measure of the relative change in period, Δ=Tmax−Tmin(Tmax+Tmin)/2, across the range of gene expression, and the absolute change (in hours): ΔT = Δ∙24 h. For PNF(1M8) we limit the increase in gene expression to 2.5 x WT value of AMAX. (DOCX) [file pcbi.1008340.s002.docx]

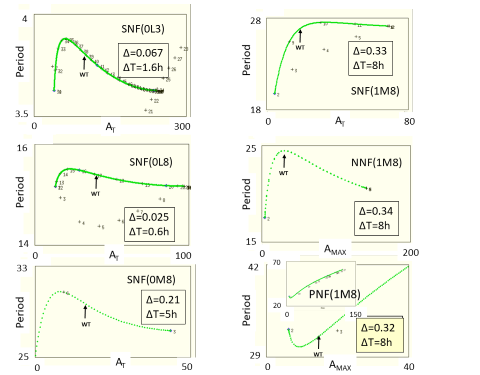


**S2 Fig. Dependence of oscillation period on level of expression of *Bmal1*, either *A*_T_ for SNF models or *A*_MAX_ for NNF and PNF models.** In the insets we record a measure of the relative change in period, $\Delta=\frac{T_{\text{max}}-T_{\text{min}}}{\left( T_{\text{max}}+T_{\text{min}} \right)/2}$ , across the range of gene expression, and the absolute change (in hours): $\Delta T=\Delta\cdot24$h. For PNF(1M8) we limit the increase in gene expression to 2.5 x WT value of *A*_MAX_.
