# Supplementary material for: Mathematical analysis of robustness of oscillations in models of the mammalian circadian clock
Source: PLoS Comput Biol. 2022 Mar 18;18(3):e1008340. doi: 10.1371/journal.pcbi.1008340 (PMC8979472; doi:10.1371/journal.pcbi.1008340)
Supplement: S3 Fig — Δ and ΔT, the relative and absolute changes in period over the range of gene expression, are defined in the legend to S2 Fig. For PNF(1M8) we limit the increase in gene expression to 2.5 x WT value of α. (DOCX) [file pcbi.1008340.s003.docx]

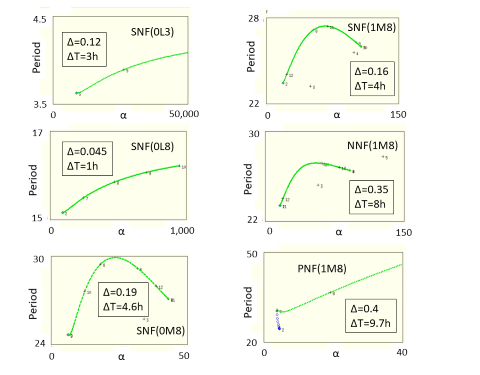


**S3 Fig. Dependence of oscillation period on level of expression of *Per*, i.e., parameter *α*.** Δ and Δ*T*, the relative and absolute changes in period over the range of gene expression, are defined in the legend to S2 Fig. For PNF(1M8) we limit the increase in gene expression to 2.5 x WT value of *α*.
