# Supplementary material for: Mathematical analysis of robustness of oscillations in models of the mammalian circadian clock
Source: PLoS Comput Biol. 2022 Mar 18;18(3):e1008340. doi: 10.1371/journal.pcbi.1008340 (PMC8979472; doi:10.1371/journal.pcbi.1008340)
Supplement: S1 Table — (DOCX) [file pcbi.1008340.s004.docx]

# S1 Table. Definitions of the dynamical variables in the models.

| Dimensionless Variable | Definition* | Physical Meaning |
| --- | --- | --- |
| *t* | $\hat{\beta}_{1}\hat{t}$ | time |
| *M* | $\frac{\hat{\alpha}_{N}\cdots\hat{\alpha}_{2}}{\hat{\beta}_{1}^{7}}\frac{\hat{M}}{\hat{K}_{\text{d}}}$ | *Per* mRNA |
| *P_i_* | $\frac{\hat{\alpha}_{N}\cdots\hat{\alpha}_{i+2}}{\hat{\beta}_{1}^{N-1-i}}\frac{\hat{P}_{i}}{\hat{K}_{\text{d}}}, i=1,\cdots,N-2$ | Cytoplasmic PER species |
| *P* | $\hat{P}/{\hat{K}_{\text{d}}}$ | Nuclear PER |
| *A* | $\hat{A}/{\hat{K}_{\text{d}}}$ | Nuclear BMAL1 |
| *R* | $\hat{R}/{\hat{K}_{\text{R}}}$ | Nuclear ROR |
| *V* | $\hat{V}/{\hat{K}_{\text{V}}}$ | Nuclear REV-ERB |

^*^All ‘hatted’ variables and parameters carry units of concentration (nM) and time (h). We have assumed that all the first-order rate constants for loss of mRNA and cytoplasmic PER species are identical: $\hat{\beta}_{1}=\hat{\beta}_{2}=\cdots=\hat{\beta}_{N-1}$.
