# Supplementary material for: Mathematical analysis of robustness of oscillations in models of the mammalian circadian clock
Source: PLoS Comput Biol. 2022 Mar 18;18(3):e1008340. doi: 10.1371/journal.pcbi.1008340 (PMC8979472; doi:10.1371/journal.pcbi.1008340)
Supplement: S2 Table — (DOCX) [file pcbi.1008340.s005.docx]

# S2 Table. Definitions of the dimensionless parameters in the models.

| Dimensionless Parameter | Definition | Meaning |
| --- | --- | --- |
| *K*_d_ | 1 | Dissociation constant of the PER:BMAL1 complex |
| *K*_A_ | ${\hat{K}_{\text{A}}}/{\hat{K}_{\text{d}}}$ | Dissociation constant of the BMAL1:Ebox complex |
| *K*_m_ | ${\hat{K}_{\text{m}}}/{\hat{K}_{\text{d}}}$ | Michaelis constant for the degradation of nuclear PER |
| *β*_max_ | ${\hat{\beta}_{\text{max}}}/\left( {\hat{\beta}_{1}\hat{K}}_{\text{d}} \right)$ | Maximum rate of degradation of nuclear PER |
| *α* | $\frac{{\hat{\alpha}_{1}\hat{\alpha}_{2}\cdots\hat{\alpha}}_{N}}{\hat{\beta}_{1}^{N}\hat{K}_{\text{d}}}$ | Maximum rate of transcription of *Per* gene |
| *A*_T_ | ${\hat{A}_{\text{T}}}/{\hat{K}_{\text{d}}}$ | Total BMAL1 concentration (bound + unbound) |
| *A*_MAX_ | ${\hat{A}_{\text{MAX}}}/{\hat{K}_{\text{d}}}$ | Maximum concentration of BMAL1 |
| *V*_MAX_ | ${\hat{V}_{\text{MAX}}}/{\hat{K}_{\text{V}}}$ | Maximum concentration of REV-ERB |
| *R*_MAX_ | ${\hat{R}_{\text{MAX}}}/{\hat{K}_{\text{R}}}$ | Maximum concentration of ROR |
| *δ* | $\hat{\delta}/{\hat{\beta}_{\text{1}}}$ | Rate constant for turnover of BMAL1, REV-ERB and ROR |
