# Supplementary material for: Mathematical analysis of robustness of oscillations in models of the mammalian circadian clock
Source: PLoS Comput Biol. 2022 Mar 18;18(3):e1008340. doi: 10.1371/journal.pcbi.1008340 (PMC8979472; doi:10.1371/journal.pcbi.1008340)
Supplement: S1 Text — (DOCX) [file pcbi.1008340.s006.docx]

# S1 Text. Goodwin’s model.

To account for observations of periodic enzyme synthesis in bacteria [1], Brian Goodwin [2, 3] presented the following model for the periodic synthesis of an enzyme Y from its mRNA X, where mRNA synthesis is inhibited by a repressor Z that is the product of the catalytic action of Y:

|  | $\frac{dX}{dT}=\alpha_{1}\frac{K^{p}}{K^{p}+Z^{p}}-\beta_{1}X$ | $\frac{dx}{dt}=\alpha\frac{1}{1+z^{p}}-x$ | (1) |
| --- | --- | --- | --- |
|  | $\frac{dY}{dT}=\alpha_{2}X-\beta_{2}Y$ | $\frac{dy}{dt}=x-y$ | (2) |
|  | $\frac{dZ}{dT}=\alpha_{3}Y-\beta_{3}Z$ | $\frac{dz}{dt}=y-z$ | (3) |

In Eq. (1), the factor $\frac{K^{p}}{K^{p}+Z^{p}}$ is the probability that the promoter region of the gene encoding X is not bound to Z, its repressor, and *α*_1_ is the maximum rate of synthesis of X by the gene. The other terms in these equations correspond to first-order rate laws for production and removal of X, Y and Z. In this tableau, Goodwin’s equations are written in two equivalent forms. On the left, the equations are written in terms of the original dimensional variables: concentrations *X*, *Y* and *Z* (nM) and time *T* (h); on the right, in terms of the ‘dimensionless’ variables $x=\frac{\alpha_{2}\alpha_{3}}{\beta_{1}^{2}}\cdot\frac{X}{K}$ , $y=\frac{\alpha_{3}}{\beta_{1}}\cdot\frac{Y}{K}$ , $z=\frac{Z}{K}$ , dimensionless time $t=\beta_{1}T$, and a dimensionless parameter $\alpha=\frac{\alpha_{1}\alpha_{2}\alpha_{3}}{K\beta_{1}^{3}}$ . In deriving these dimensionless equations, we have assumed (as have all authors in the past) that $\beta_{1}=\beta_{2}=\beta_{3}$, which serves to maximize the oscillatory potential of the model [4, 5]. In Goodwin’s version of a three-component negative-feedback loop, the repression of gene transcription by Z is modeled by a Hill function with exponent *p*. Underlying this function is the supposition that the gene encoding X is turned off when *p* molecules of Z bind cooperatively to its promoter region (or, equivalently, when *p* molecules of Z bind cooperatively to an activator of gene transcription and shut it off).

A problem with Goodwin’s model. J.S. Griffith [6] was first to point out that Goodwin’s equations (1)-(3) admit oscillatory solutions only if $p>\text{sec}^{3}\left( \pi/3 \right)=8$, a very restrictive condition, because in experimental studies it is rare that more than 3 or 4 protein molecules bind cooperatively to DNA regulatory sequences [7]. This condition becomes even more restrictive if $\beta_{1}\neq\beta_{2}\neq\beta_{3}$ [4].

One solution: a longer feedback loop. The restriction *p* > 8 can be ameliorated by lengthening the feedback loop: if *n* = number of variables in the feedback loop, then the necessary condition for oscillations becomes $p>\text{sec}^{n}\left( \pi/n \right)$. For example, for *n* = 8, the condition is *p* > 1.88. Longer loops (*n* > 3) correspond to inserting more than one intermediate (say, Y_0_, Y_1_, …, Y*_n_*_−3_) between X (mRNA) and Z (feedback component). This is quite reasonable, considering that PER protein has multiple phosphorylation sites [8]. Each intermediate, Y*_j_*, then denotes cytoplasmic PER phosphorylated on *j* sites, *j* = 0, 1, …, *J*. Eventually, the fully phosphorylated form, Y*_J_*, is transported into the nucleus and becomes Z. In this case, Goodwin’s dimensionless differential equations become Eqs. (4)-(7).

|  | $\frac{dx}{dt}=\alpha\frac{1}{1+z^{p}}-x$ | (4) |
| --- | --- | --- |
|  | $\frac{dy_{0}}{dt}=x-y_{0}$ | (5) |
|  | $\frac{dy_{j}}{dt}=y_{j-1}-y_{j}, for j=1,\ldots,J$ | (6) |
|  | $\frac{dz}{dt}=y_{J}-z$ | (7) |

Exactly the same equations can be derived by assuming a distributed time lag between *x* and *z* [9]:

|  | $z\left( t \right)=\int_{-\infty}^{t} G_{J+1}\left( t-s \right)x\left( s \right)ds, where G_{j}\left( u \right)=u^{j}e^{-u}/j!$ | (8) |
| --- | --- | --- |

Introduce the new variables,

|  | $y_{j}\left( t \right)=\int_{-\infty}^{t} G_{j}\left( t-s \right)x\left( s \right)ds, for j=0,1,\ldots,J$ | (9) |
| --- | --- | --- |

For *j* = 0, we have Eq. (5) for *dy*_0_/*dt*. For *j* ≥ 1, *G_j_*(0) = 0 and d*G_j_*(*u*)/d*u* = *G_j_*_−1_(*u*)− *G_j_*(*u*); so we have Eq. (6) for *dy*_j_/*dt* for $j=1,\ldots,J$and Eq. (7) for *dz*/*dt*.

A second solution: Michaelis-Menten degradation of Z. In 1982 Bliss, Painter and Marr [10] proposed to replace the first-order degradation of Z, $-\beta_{3}Z,$ by a Michaelis-Menten rate law, $-\beta_{3}Z/\left( K_{\text{m}}+Z \right),$ where $K_{\text{m}}$ is the ‘Michaelis’ constant of the enzyme-catalyzed reaction and $\beta_{3}$ is the ‘*V*_max_’ of the reaction. With this change, the Goodwin model can exhibit limit cycle oscillations even for *p* = 1 [10]. The substitution of Michaelis-Menten rate laws for the first-order kinetic terms in Eqs. (1)-(3) has been exploited by many authors [11-13] to increase the robustness of their models of circadian rhythms.

# References

1. Masters M, Donachie WD. Repression and the control of cyclic enzyme synthesis in Bacillus subtilis. Nature. 1966;209(5022): 476-9. doi: 10.1038/209476a0.

2. Goodwin BC. Oscillatory behavior in enzymatic control processes. Adv Enzyme Regul. 1965;3: 425-38.

3. Goodwin BC. An entrainment model for timed enzyme syntheses in bacteria. Nature. 1966;209(5022): 479-81. doi: 10.1038/209479a0.

4. Tyson JJ, Othmer HG. The Dynamics of Feedback Control Circuits in Biochemical Pathways. In Progress in Theoretical Biology: Elsevier; 1978. p. 1-62.

5. Rapp P. Analysis of Biochemical Phase-Shift Oscillators by a Harmonic Balancing Technique. J Math Biol. 1976;3(3-4): 203-24. doi: 10.1007/Bf00275057.

6. Griffith JS. Mathematics of cellular control processes. I. Negative feedback to one gene. J Theor Biol. 1968;20(2): 202-8. doi: 10.1016/0022-5193(68)90189-6.

7. Gonze D, Abou-Jaoude W. The Goodwin model: behind the Hill function. PLoS One. 2013;8(8): e69573. doi: 10.1371/journal.pone.0069573.

8. Vanselow K, Vanselow JT, Westermark PO, Reischl S, Maier B, Korte T, et al. Differential effects of PER2 phosphorylation: molecular basis for the human familial advanced sleep phase syndrome (FASPS). Genes Dev. 2006;20(19): 2660-72. doi: 10.1101/gad.397006.

9. MacDonald N. Time Lags in Biological Models. 1 ed: Springer-Verlag Berlin Heidelberg; 1978. VIII, 114 p.

10. Bliss RD, Painter PR, Marr AG. Role of feedback inhibition in stabilizing the classical operon. J Theor Biol. 1982;97(2): 177-93. doi: 10.1016/0022-5193(82)90098-4.

11. Goldbeter A. A model for circadian oscillations in the Drosophila period protein (PER). Proc R Soc Lond B. 1995;261(1362): 319-24. doi: 10.1098/rspb.1995.0153.

12. Kurosawa G, Iwasa Y. Saturation of enzyme kinetics in circadian clock models. J Biol Rhythms. 2002;17(6): 568-77. doi: 10.1177/0748730402238239.

13. Gonze D, Bernard S, Waltermann C, Kramer A, Herzel H. Spontaneous synchronization of coupled circadian oscillators. Biophys J. 2005;89(1): 120-9. doi: 10.1529/biophysj.104.058388.
