# Supplementary material for: Mathematical analysis of robustness of oscillations in models of the mammalian circadian clock
Source: PLoS Comput Biol. 2022 Mar 18;18(3):e1008340. doi: 10.1371/journal.pcbi.1008340 (PMC8979472; doi:10.1371/journal.pcbi.1008340)
Supplement: S2 Text — (DOCX) [file pcbi.1008340.s007.docx]

# S2 Text. Kim & Forger’s extended models.

In addition to the SNF model, Kim & Forger [1] proposed two extended models, in which the core negative feedback loop involving PER and BMAL1 is supplemented with (either) an additional negative feedback from REV-ERB on transcription of the *Bmal1* gene (or) an additional positive feedback from ROR on transcription of the *Bmal1* gene. Both extended models include the ODEs of the core SNF model.

Kim-Forger NNF Model. Equations (1)-(4) of the main text, plus

|  | $\frac{dV}{dt}=\delta\left( V_{\text{max}}\frac{A_{\mathrm{free}}}{A_{\text{T}}}-V \right)$ | (1) |
| --- | --- | --- |
|  | $\frac{dA_{T}}{dt}=\delta\left( \frac{V_{\text{o}}}{V}-A_{T} \right)$ | (2) |

where *V* is the (scaled) concentration of REV-ERB, *V*_max_ is the maximum achievable concentration of REV-ERB, *V*_o_ is the REV-ERB concentration that would result in *A*_T_ = 1 at steady state, and *δ* is a rate constant that sets the time scale for the feedback loop.

Kim-Forger PNF Model. Equations (1)-(4) of the main text, plus

|  | $\frac{dR}{dt}=\delta\left( R_{\text{max}}\frac{A_{\mathrm{free}}}{A_{\text{T}}}-V \right)$ | (3) |
| --- | --- | --- |
|  | $\frac{dA_{T}}{dt}=\delta\left( \frac{R}{R_{\text{o}}}-A_{T} \right)$ | (4) |

where *R* is the (scaled) concentration of ROR, and *R*_max_, *R*_o_ and *δ* are defined similarly as in the NNF equations. For simulations of the NNF and PNF models, Kim & Forger chose *δ* = 0.2, *V*_max_ = *R*_max_ = 5, and they adjusted *V*_o_ and *R*_o_ to make the NNF and PNF models have the same average activator concentration, <*A*_T_>, as the SNF model.

# Reference

1. Kim JK, Forger DB. A mechanism for robust circadian timekeeping via stoichiometric balance. Mol Syst Biol. 2012;8: 630. doi: 10.1038/msb.2012.62.
