# Supplementary material for: Mathematical analysis of robustness of oscillations in models of the mammalian circadian clock
Source: PLoS Comput Biol. 2022 Mar 18;18(3):e1008340. doi: 10.1371/journal.pcbi.1008340 (PMC8979472; doi:10.1371/journal.pcbi.1008340)
Supplement: S3 Text — (DOCX) [file pcbi.1008340.s008.docx]

# S3 Text. Parameter optimization for models with saturating degradation of nuclear PER.

We used MATLAB’s simulated annealing method (‘simulannealbnd’) to optimize the parameters of SNF(0M8), SNF(1M8), NNF(1M8) and PNF(1M8) models within physiologically reasonable ranges, as indicated below. The optimization criteria we used for each model and the corresponding cost functions are also given below. The model outputs used in the cost functions were obtained by simulating the model with the corresponding parameter set. Specifically, we used MATLAB’s stiff ODE solver (‘ode15s’) to generate simulated trajectories with final time = 2,000. For each trajectory we analyzed the segment between times 1,800 and 2,000 to obtain the output quantities of interest, such as period, max(*P*_tot_) and amp(*P*_tot_). We performed simulated annealing multiple times, starting from 1,100 random initial guesses of parameter values generated by the Latin hypercube method applied to log-uniform distributions of the parameters over the same ranges that bound the optimization. In a small fraction of cases for each model, the optimization aborted and generated no results. We present below the most noteworthy patterns based on all the results we obtained.

Optimization of SNF(0M8)

Criteria:

1. max(*P*_tot_) is minimized, in order to select parameters sets with the largest values of $\hat{K}_{\text{d}}$.
2. Relative amplitude of *P*_tot_ > 0.5, in order to select parameter sets that generate robust oscillation.

Cost function:

|  | $C=\frac{\max\left( P_{\mathrm{tot}} \right)}{75}$  $+\left( \frac{0.5}{\mathrm{amp}\left( P_{\mathrm{tot}} \right)}+\left( \frac{\mathrm{amp}\left( P_{\mathrm{tot}} \right)}{0.5}-1 \right)^{2} \right)\left( 1-H\left( \mathrm{amp}\left( P_{\mathrm{tot}} \right)-0.5 \right) \right)$  $+ H\left( \mathrm{amp}\left( P_{\mathrm{tot}} \right)-0.5 \right)$ |  |
| --- | --- | --- |

The second criterion was implemented in a complicated form to penalize oscillations of small amplitude and avoid getting stuck with non-oscillating parameters.

Range of parameters:

$$A_{T}\in\left[ {10}^{-2},{10}^{2} \right],\alpha\in\left[ {10}^{-2},{10}^{3} \right],K_{m}\in\left[ 1,{10}^{2} \right],\beta_{\mathrm{MAX}}\in[{10}^{-2},{10}^{3}]$$

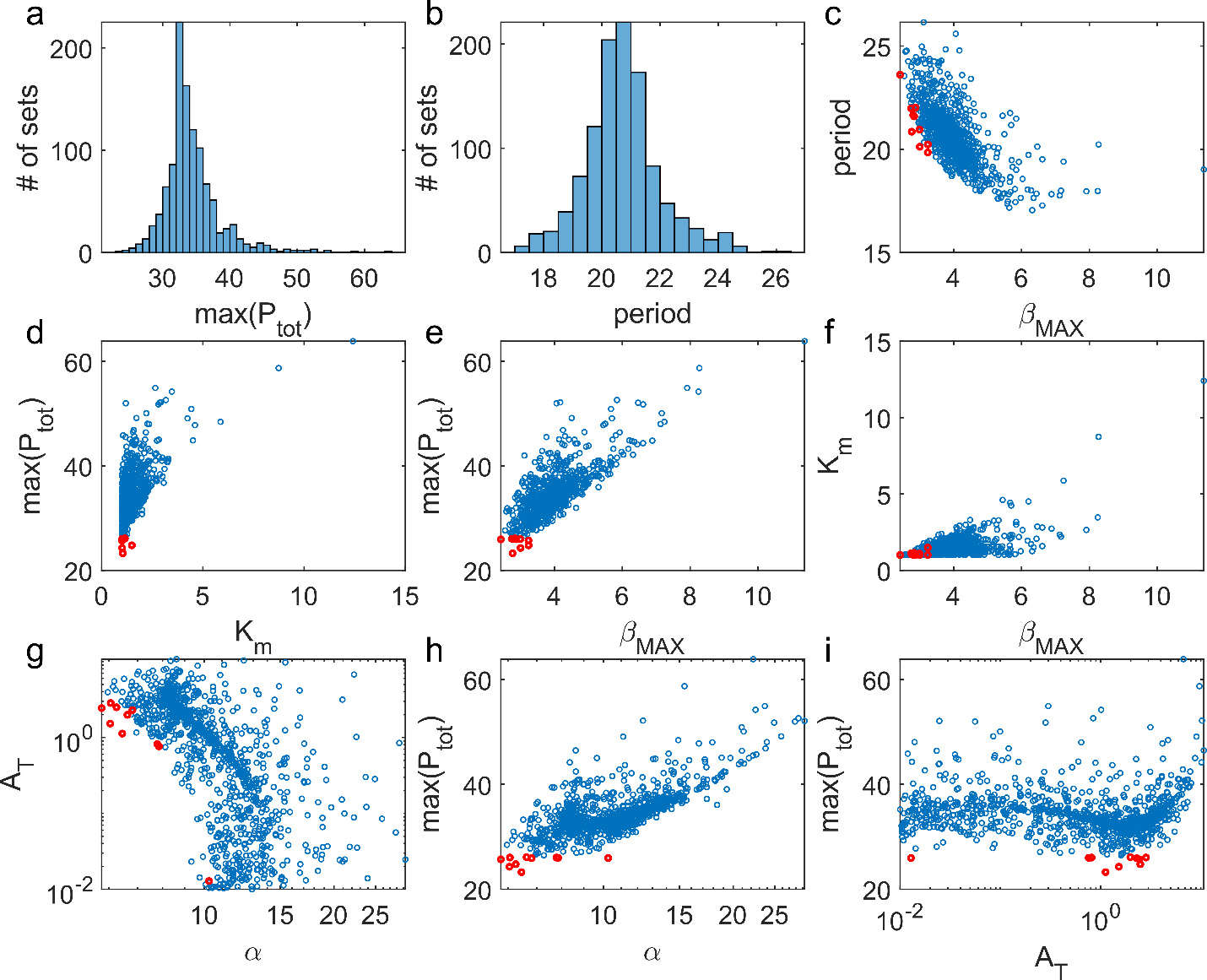


**Fig 1. Notable patterns in optimization results for SNF(0M8)**. Blue: 1100 parameter sets obtained through optimization are plotted in each panel. Red: top 10 parameter sets, i.e., those with the smallest values of max(*P*_tot_).

Main findings:

1. max(*P*_tot_) = 34.3 ± 4.0 (Fig 1a). Period = 20.7 ± 1.3 (Fig 1b).
2. Period is strongly negatively correlated with *β*_max_ (Fig 1c), but not other parameters (not shown). This is not surprising, since smaller values of *β*_max_ mean a longer time delay for degrading nuclear PER and reinstituting *Per* gene transcription. The top 10 sets generate intermediate periods between 20 and 24 (Fig 1c).
3. Low max(*P*_tot_) requires low *K*_m_ and *β*_max_ (Fig 1d-f). Lower values in both parameters slow down PER degradation, introduce time delay and increase robustness of oscillation (allowing oscillation to happen for larger values of $\hat{K}_{\text{d}}$).
4. *α* and *A*_T_ are negatively correlated (Fig 1g), which is opposite to the trend shown in the main text (Fig 6a) for SNF(0L8), although we are comparing different types of diagrams.
5. max(*P*_tot_) is positively correlated to *α* (Fig 1h, as expected) but not to *A*_T_ (Fig 1i).
6. The range of optimal values of *α* (1—30) is much smaller than the range of *A*_T_ (0.01—10) (Fig 1g). Because its rate of degradation saturates at high concentration, the level of PER in the nucleus tends to stay high for an extended period of time, which places a constraint on the rate of expression of the *Per* gene.

Optimization of SNF(1M8)

Criteria:

1. max(*P*_tot_) is minimized, in order to select parameters sets with the largest values of $\hat{K}_{\text{d}}$.
2. Relative amplitude of *P*_tot_ > 0.5, in order to select parameter sets that generate robust oscillation.

Cost function:

|  | $C=\frac{\max\left( P_{\mathrm{tot}} \right)}{75}$  $+\left( \frac{0.5}{\mathrm{amp}\left( P_{\mathrm{tot}} \right)}+\left( \frac{\mathrm{amp}\left( P_{\mathrm{tot}} \right)}{0.5}-1 \right)^{2} \right)\left( 1-H\left( \mathrm{amp}\left( P_{\mathrm{tot}} \right)-0.5 \right) \right)$  $+ H\left( \mathrm{amp}\left( P_{\mathrm{tot}} \right)-0.5 \right)$ |  |
| --- | --- | --- |

The second criterion was implemented in a complicated form to penalize oscillations of small amplitude and avoid getting stuck with non-oscillating parameters.

Range of parameters:

$$A_{T}\in\left[ {10}^{-2},{10}^{2} \right],K_{A}\in\left[ 1,{10}^{2} \right],\alpha\in\left[ {10}^{-2},{10}^{3} \right],K_{m}\in\left[ 1,{10}^{2} \right],\beta_{\mathrm{MAX}}\in[{10}^{-2},{10}^{3}]$$

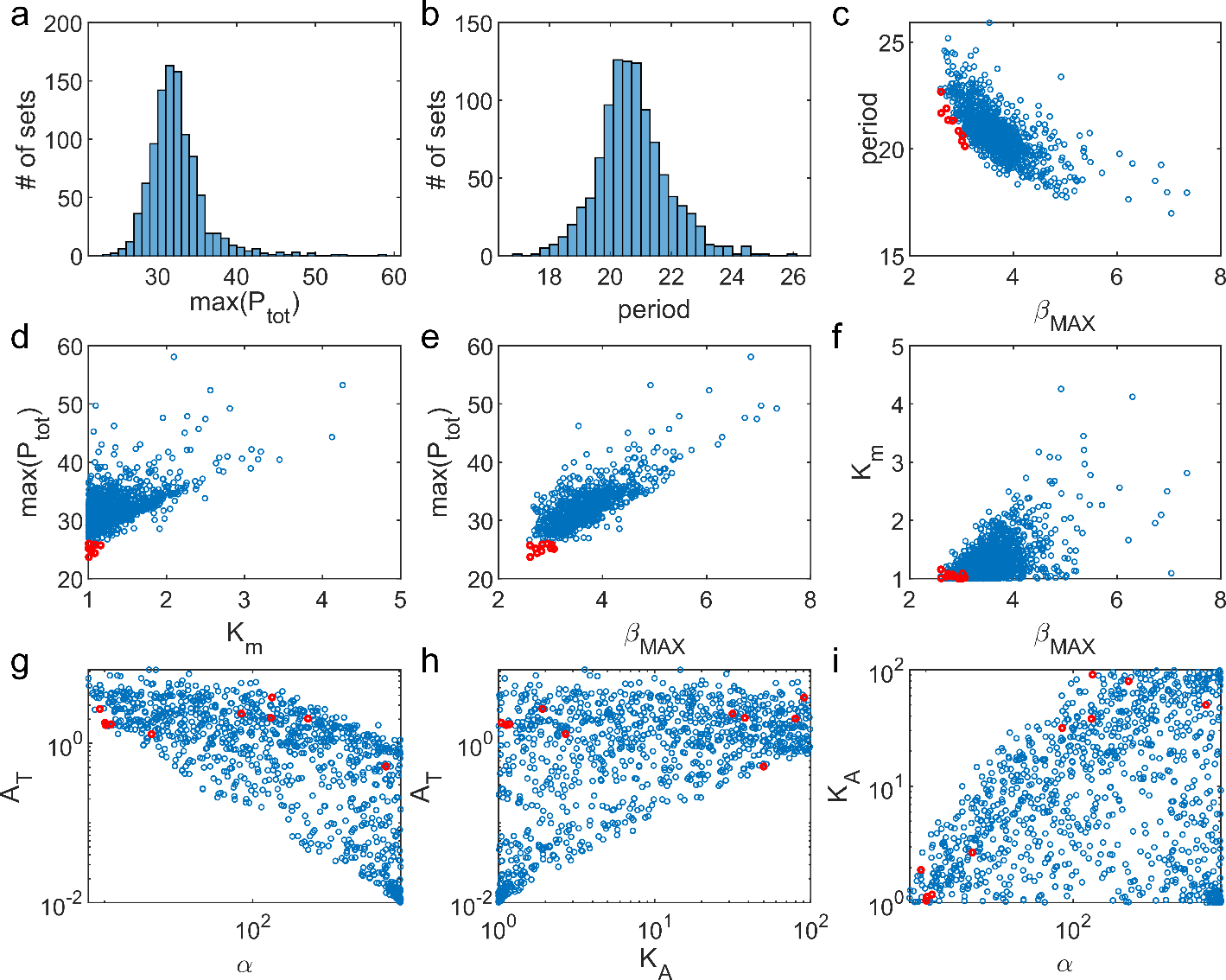


**Fig 2. Notable patterns in optimization results for SNF(1M8)**. Blue: 1013 parameter sets obtained through optimization are plotted in each panel. Red: top 10 parameter sets, i.e., those with the smallest values of max(*P*_tot_).

Main findings:

1. max(*P*_tot_) = 32.3 ± 3.5 (Fig 2a). Period = 20.7 ± 1.1 (Fig 2b). The distributions are quite similar to SNF(0M8) in Fig 1a, b.
2. Period is strongly negatively correlated with *β*_max_ (Fig 2c), but not other parameters. This is not surprising, since smaller values of *β*_max_ mean a longer time delay for degrading nuclear PER and reinstituting *Per* gene transcription. The top 10 sets generate intermediate periods between 20 and 23 (Fig 2c).
3. Low max(*P*_tot_) requires low *K*_m_ and *β*_max_ (Fig 2d-f). Lower values in both parameters slow down PER degradation, introduce time delay and increase robustness of oscillation (allowing oscillation to happen for larger values of $\hat{K}_{\text{d}}$).
4. *α* and *A*_T_ are negatively correlated (Fig 2g). This result is opposite to the trend in L models (see main text Fig 6b, although we are comparing different types of diagrams).
5. *A*_T_ must be greater than ~ *K*_A_/100 (Fig 2h). The top 10 sets are concentrated in a narrow range of *A*_T_ between 1 and 5 (Fig 2h).
6. *α* and *K*_A_ are positively correlated, and *α* must be greater than $\sim10\sqrt{K_{A}}$ (Fig 2i), presumably to have sufficiently rapid transcription of *Per* gene as *K*_A_ increases. Also, for small values of *A*_T_, *α* can be much larger in SNF(1M8) compared to SNF(0M8), because the rate of *Per* transcription is more restricted at low *A*_T_ in rate law 1 compared to rate law 0.

Optimization of NNF(1M8)

Criteria:

1. max(*P*_tot_) is minimized in order to select parameters sets with the largest values of $\hat{K}_{\text{d}}$.
2. Relative amplitude of *P*_tot_ > 0.5, in order to select parameter sets that generate robust oscillation.
3. Relative amplitude of *A*_T_ > 0.2, because experimental data show ~20% amplitude in BMAL1 oscillation [1].
4. max(*V*) < 10, in order that $\hat{K}_{\text{V}}$, the equilibrium dissociation constant for REV-ERB binding to the promotor of the *Bmal1* gene, is not too small.
5. max(*A*_T_) / max(*P*_tot_) as close to 1 as possible; this criterion is introduced because without it we often ended up with parameter sets for which max(*A*_T_) / max(*P*_tot_) is very small. However, experimental data show that the peak levels of PER and BMAL1 are comparable [1].

Cost function:

|  | $C=\frac{\max\left( P_{\mathrm{tot}} \right)}{75}$  $+\left( \frac{0.5}{\mathrm{amp}\left( P_{\mathrm{tot}} \right)}+\left( \frac{\mathrm{amp}\left( P_{\mathrm{tot}} \right)}{0.5}-1 \right)^{2} \right)\left( 1-H\left( \mathrm{amp}\left( P_{\mathrm{tot}} \right)-0.5 \right) \right)+ H\left( \mathrm{amp}\left( P_{\mathrm{tot}} \right)-0.5 \right)$  $+\left( \frac{0.2}{\mathrm{amp}\left( A_{T} \right)}+\left( \frac{\mathrm{amp}\left( A_{T} \right)}{0.2}-1 \right)^{2} \right)\left( 1-H\left( \mathrm{amp}\left( A_{T} \right)-0.2 \right) \right)+H\left( \mathrm{amp}\left( A_{T} \right)-0.2 \right)$  $+\left( \frac{\max\left( V \right)}{10}-1 \right)^{2}H\left( \max\left( V \right)-10 \right)$  $+\left\vert\frac{\max\left( P_{\mathrm{tot}} \right)}{\max\left( A_{T} \right)}-1 \right\vert$ |  |
| --- | --- | --- |

Criteria 2 and 3 above were implemented with functions taking a form similar to Criterion 2 in the SNF model.

Range of parameters:

$$\delta\in\left[ {10}^{-2},{10}^{2} \right],A_{\mathrm{MAX}}\in\left[ {10}^{-2},{10}^{3} \right],R_{\mathrm{MAX}}\in\left[ {10}^{-2},{10}^{2} \right],$$

$$K_{A}\in\left[ 1,{10}^{2} \right],\alpha\in\left[ {10}^{-2},{10}^{3} \right],K_{m}\in\left[ 1,{10}^{2} \right],\beta_{\mathrm{MAX}}\in[{10}^{-2},{10}^{3}]$$

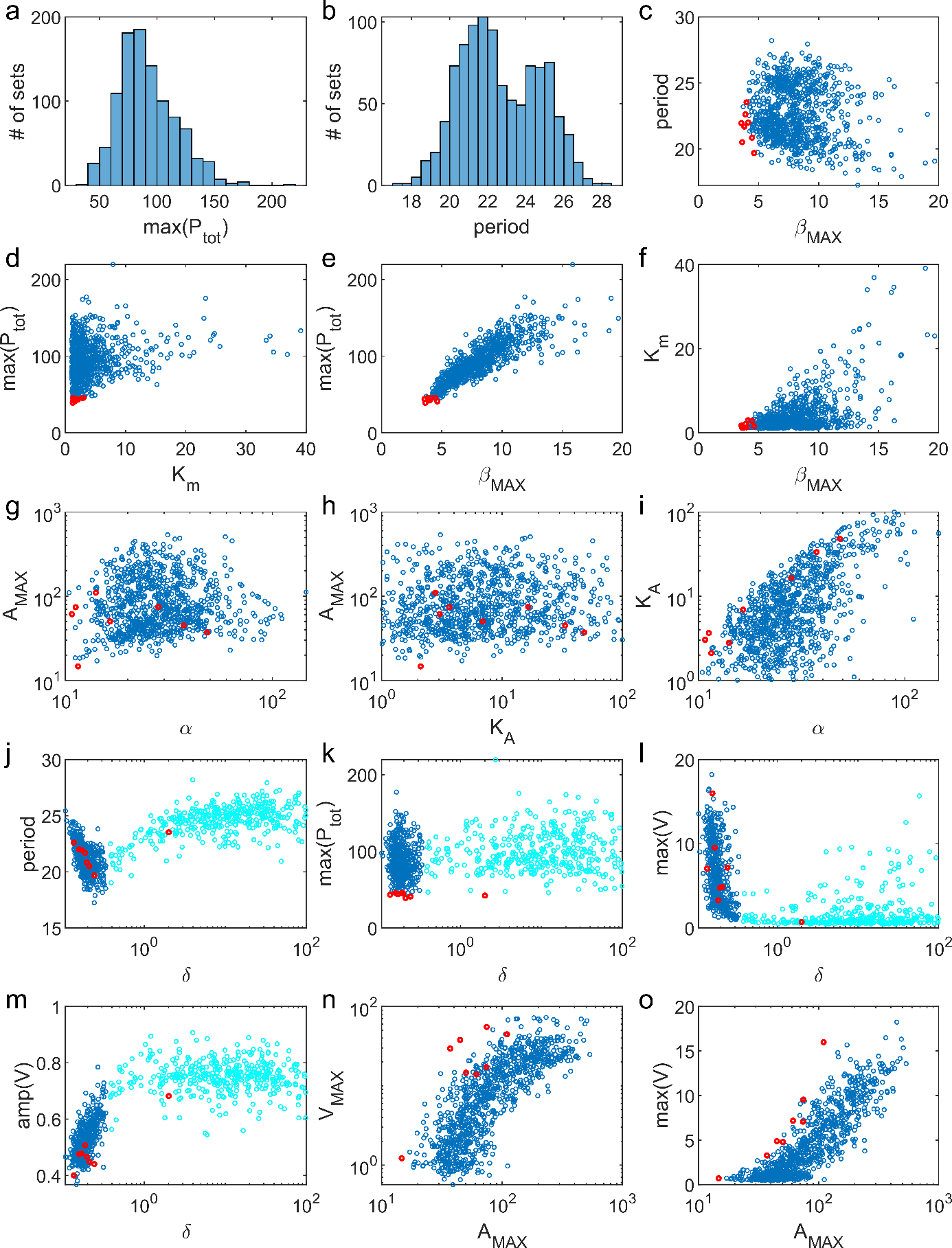


**Fig 3.** **Notable patterns in optimization results for NNF(1M8)**. Blue: 1011 parameter sets obtained through optimization are plotted in each panel. Cyan in (j)-(m): 423 parameter sets with *δ* > 0.35. (The *δ* > 0.35 sets are not highlighted in the other panels because they are mixed with the *δ* < 0.35 group.) Red: top 10 parameter sets (with smallest values of max(*P*_tot_)).

Main findings:

1. max(*P*_tot_) = 91.7 ± 24.5 (Fig 3a). Period = 22.7 ± 2.1 (bimodal, Fig 3b).
2. Period is not strongly correlated with *β*_max_ (Fig 3c), which is surprising. But it is strongly correlated with *δ* (Fig 3j).
3. Low max(*P*_tot_) requires low *K*_m_ and *β*_max_ (Fig 3d-f), but not as strongly as in SNF (Fig 2d-f). Note that Fig 3d-f and Fig 2d-f have very different axis ranges.
4. Unlike SNF models, *α* and *A*_MAX_ are no longer correlated (Fig 3g). Neither are *A*_MAX_ and *K*_A_ (Fig 3h). But *α* and *K*_A_ remain positively correlated (Fig 3i).
5. With regard to the time-scale parameter, *δ*, there appear to be two clusters, separated by a value of ~0.35 (Fig 3j, k). The majority of top sets (red) have *δ* < 0.35. In comparison to the *δ* < 0.35 cluster, the *δ* > 0.35 cluster is associated with *V*(*t*) with smaller maximum values (Fig 3l) yet larger amplitudes (Fig 3m).
6. *A*_MAX_ is positively correlated with *V*_MAX_ (Fig 3n). Top 10 sets are associated with nearly the highest ratio of *V*_MAX_ to *A*_MAX_. Consistently, these top sets generate the highest max(*V*) relative to the value of *A*_MAX_ (Fig 3o). Additionally, they tend to generate low amplitude of *V*(*t*) in the *δ* < 0.35 cluster. Presumably, a high ratio of *V*_MAX_ to *A*_MAX_ enhances the inhibition of BMAL1 expression by REV-ERB, which can help suppress BMAL1 for a longer time even if PER does not bind BMAL1 as tightly (i.e., larger $\hat{K}_{d}$ or lower max(*P*_tot_)).

Optimization of PNF(1M8)

Criteria:

1. max(*P*_tot_) is minimized, i.e., $\hat{K}_{\text{d}}$ is maximized.
2. Relative amplitude of *P*_tot_ > 0.5, i.e., oscillations are robust.
3. Relative amplitude of *A*_T_ > 0.2, as for NNF.
4. max(*R*) < 5, so that $\hat{K}_{\text{R}}$, the equilibrium dissociation constant for ROR binding to the promotor of the *Bmal1* gene, is not too small.
5. max(*A*_T_) / max(*P*_tot_) as close to 1 as possible, as for NNF.

Cost function:

|  | $C=\frac{\max\left( P_{\mathrm{tot}} \right)}{75}$  $+\left( \frac{0.5}{\mathrm{amp}\left( P_{\mathrm{tot}} \right)}+\left( \frac{\mathrm{amp}\left( P_{\mathrm{tot}} \right)}{0.5}-1 \right)^{2} \right)\left( 1-H\left( \mathrm{amp}\left( P_{\mathrm{tot}} \right)-0.5 \right) \right)+ H\left( \mathrm{amp}\left( P_{tot} \right)-0.5 \right)$  $+\left( \frac{0.2}{\mathrm{amp}\left( A_{T} \right)}+\left( \frac{\mathrm{amp}\left( A_{T} \right)}{0.2}-1 \right)^{2} \right)\left( 1-H\left( \mathrm{amp}\left( A_{T} \right)-0.2 \right) \right)+H\left( \mathrm{amp}\left( A_{T} \right)-0.2 \right)$  $+\left( \frac{\max\left( V \right)}{5}-1 \right)^{2}H\left( \max\left( R \right)-5 \right)$  $+\left\vert\frac{\max\left( P_{\mathrm{tot}} \right)}{\max\left( A_{T} \right)}-1 \right\vert$ |  |
| --- | --- | --- |

Similar to the cost function for the NNF model.

Range of parameters:

$$\delta\in\left[ {10}^{-2},{10}^{2} \right],A_{\mathrm{MAX}}\in\left[ {10}^{-2},{10}^{3} \right],R_{\mathrm{MAX}}\in\left[ {10}^{-2},{10}^{2} \right], \varepsilon\in\left[ {10}^{-4},{10}^{-1} \right]$$

$$K_{A}\in\left[ 1,{10}^{2} \right],\alpha\in\left[ {10}^{-2},{10}^{3} \right],K_{m}\in\left[ 1,{10}^{2} \right],\beta_{\mathrm{MAX}}\in[{10}^{-2},{10}^{3}]$$

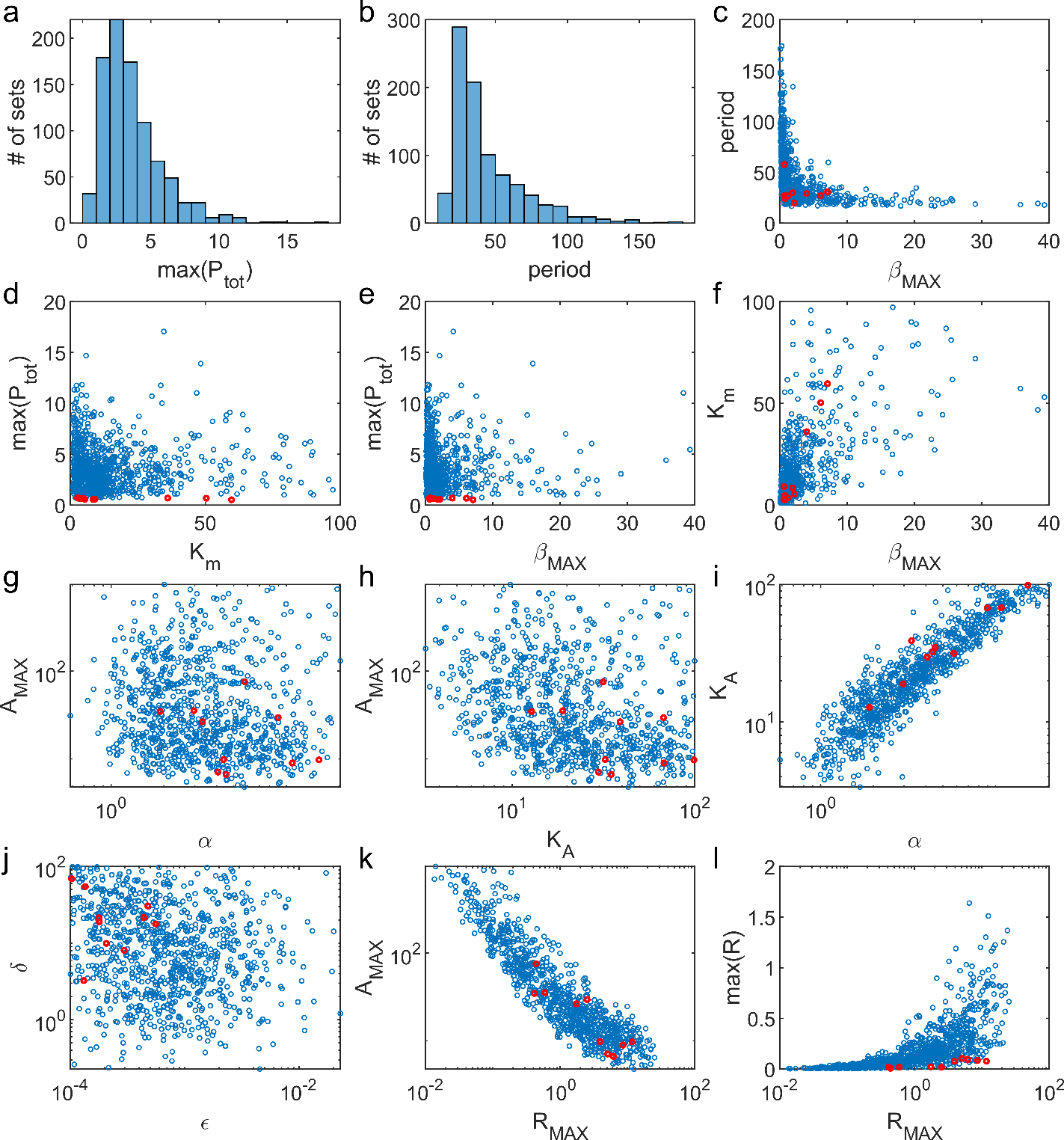


**Fig 4.** **Notable patterns in optimization results for PNF(1M8)**. Blue: 898 parameter sets obtained through optimization are plotted in each panel. Red: top 10 parameter sets (with smallest values of max(*P*_tot_)).

Main findings:

1. max(*P*_tot_) = 3.60 ± 2.21 (Fig 4a), Period = 43.9 ± 25.4 (Fig 4b).
2. Period is strongly negatively correlated with *β*_max_ (Fig 4c), as in SNF model (Fig 2c).
3. Low max(*P*_tot_) still requires low *β*_max_ (Fig 4e), but is less dependent on *K*_m_ (Fig 4d).
4. Like the NNF model, *α* and *A*_MAX_ are no longer correlated (Fig 4g), nor are *A*_MAX_ and *K*_A_ (Fig 4h). But *α* and *K*_A_ are strongly positively correlated (Fig 4i).
5. For most of the optimized parameter sets, the time-scale parameter *δ* > 1 (Fig 4j), and in all cases *ε* << 1 (Fig 4j). Note that *ε* is the ‘background’ rate of *Per* gene transcription when *R*(*t*) = 0. The parameter *ε* was introduced to avoid the trivial steady state, which often prevents the simulation to generate the oscillatory solution.
6. *A*_MAX_ and *R*_MAX_ are strongly negatively correlated (Fig 4k), opposite to the relation between *A*_MAX_ and *V*_MAX_ in the NNF model (Fig 3n). Since BMAL1 and ROR enhance the expression of each other, this negative relation probably helps stabilize the BMAL1 level (recall that the best sets for the SNF model have *A*_T_ in a narrow range).
7. Although the parameter *R*_MAX_ and the output max(*R*) are positively correlated as expected, the top sets are associated with max(*R*) << 1 (Fig 4l). Recall that the non-dimensionalized dissociation constant between ROR and the *Bmal1* gene is 1. max(*R*) << 1 indicates that robust oscillation is favored by ROR levels far below levels that saturate binding to the *Bmal1* gene, presumably by maintaining sensitivity of the auxiliary positive feedback loop.

# Reference

1. Narumi R, Shimizu Y, Ukai-Tadenuma M, Ode KL, Kanda GN, Shinohara Y, et al. Mass spectrometry-based absolute quantification reveals rhythmic variation of mouse circadian clock proteins. Proceedings of the National Academy of Sciences of the United States of America. 2016;113(24):E3461-E7. doi: 10.1073/pnas.1603799113.
