# Supplementary material for: Mathematical analysis of robustness of oscillations in models of the mammalian circadian clock
Source: PLoS Comput Biol. 2022 Mar 18;18(3):e1008340. doi: 10.1371/journal.pcbi.1008340 (PMC8979472; doi:10.1371/journal.pcbi.1008340)
Supplement: S4 Text — (DOCX) [file pcbi.1008340.s009.docx]

# S4 Text. Deriving the rate laws for *Per* transcription.

BMAL1:CLOCK binds to many E-box sequences throughout the mammalian genome, and PER:CRY binds to both free and E-box-bound BMAL1:CLOCK complexes. Let E*_i_*, *i* = 1, …, Ω, denote all the E-box sequences that bind all four proteins BMAL1, CLOCK, PER and CRY, where Ω ≈ 1500 according to ChIP-seq data [1]. The total concentrations of BMAL1:CLOCK (A) and PER:CRY (P) dimers are:

|  | | ${[\text{A}]}_{\text{T}}=\left[ \text{A} \right]+\left[ \text{A:P} \right]+\sum_{i=1}^{\Omega} \left( \left[ \text{A:}\text{E}_{i} \right]+\left[ \text{P:A:}\text{E}_{i} \right] \right)$ | | (1) |
| --- | --- | --- | --- | --- |
|  | ${[\text{P}]}_{\text{T}}=\left[ \text{P} \right]+\left[ \text{A:P} \right]+\sum_{i=1}^{\Omega} \left[ \text{P:A:}\text{E}_{i} \right]$ | | (2) | |

We are assuming that the synthesis and degradation of proteins are much slower reactions than the association and dissociations of proteins in a complex, so the total amounts of A and P in the system can be treated as constants on the time scale of the binding and unbinding reactions.

Because the total number of E-boxes (~1500) is considerably less than the total number of BMAL1:CLOCK and PER:CRY complexes (~25,000 and ~30,000, respectively [2]), we can reasonably neglect the E-box-bound forms of the protein complexes, and assume that

|  | ${[\text{A}]}_{\text{T}}\approx\left[ \text{A} \right]+\left[ \text{A:P} \right]$ | (3) |
| --- | --- | --- |
|  | ${[\text{P}]}_{\text{T}}\approx\left[ \text{P} \right]+\left[ \text{A:P} \right]$ | (4) |

To derive an expression for the rate of *Per* transcription, we need to estimate the fraction of E-boxes bound to BMAL1:CLOCK but not to PER:CRY, i.e., [A:E*_p_*]/[E*_p_*]_T_, where *p* is the index corresponding to E-boxes driving *Per* gene expression. To this end, we consider the equilibrium binding reactions:

|  | $\text{A + P}\underset{\leftrightarrow}{K_{\text{dAP1}}}\text{A:P}$ | (5) |
| --- | --- | --- |
|  | $\text{A + }\text{E}_{p}\underset{\leftrightarrow}{K_{\text{dAE1}}}\text{A:}\text{E}_{p}(active PER gene)$ | (6) |
|  | $\text{A:}\text{E}_{p}+\text{P}\underset{\leftrightarrow}{K_{\text{dAP2}}}\text{P:A:}\text{E}_{p}$ | (7) |
|  | $\text{A:P}+\text{E}_{p}\underset{\leftrightarrow}{K_{\text{dAE2}}}\text{P:A:}\text{E}_{p}$ | (8) |

The equilibrium dissociation constants of Reactions (5)-(8) are defined by

|  | $K_{\text{dAP1}}=\frac{\left[ \text{A} \right][\text{P}]}{[\text{A:P}]},K_{\text{dAE1}}=\frac{\left[ \text{A} \right][\text{E}_{p}]}{[\text{A:}\text{E}_{p}]},K_{\text{dAP2}}=\frac{\left[ \text{P} \right][\text{A:}\text{E}_{p}]}{[\text{P:A:}\text{E}_{p}]},K_{\text{dAE2}}=\frac{\left[ \text{A:P} \right][\text{E}_{p}]}{[\text{P:A:}\text{E}_{p}]}$ | (9) |
| --- | --- | --- |

The principle of detailed balance at equilibrium requires that

|  | $\underset{K_{dAE1}}{\underbrace{\frac{\left[ \text{A} \right][\text{E}_{p}]}{[\text{A:}\text{E}_{p}]}}}\underset{K_{dAP2}}{\underbrace{\frac{\left[ \text{P} \right][\text{A:}\text{E}_{p}]}{[\text{P:A:}\text{E}_{p}]}}}=\underset{K_{dAP1}}{\underbrace{\frac{\left[ \text{A} \right][\text{P}]}{[\text{A:P}]}}}\underset{K_{dAE2}}{\underbrace{\frac{\left[ \text{A:P} \right][\text{E}_{p}]}{[\text{P:A:}\text{E}_{p}]}}}$ | (10) |
| --- | --- | --- |

That is, the dissociation constants for Reactions (5)-(8) satisfy

|  | $\frac{K_{\text{dAE1}}}{K_{\text{dAE2}}}=\frac{K_{\text{dAP1}}}{K_{\text{dAP2}}}$ | (11) |
| --- | --- | --- |

Taking Eq. (11) into account, we are left with only three independent chemical equilibrium equations. One of these equations is

|  | $K_{\text{dAP1}}=\frac{\left[ \text{A} \right][\text{P}]}{[\text{A:P}]}\approx\frac{\left( \left[ \text{A} \right]_{\text{T}}-[\text{A:P}] \right)\left( \left[ \text{P} \right]_{\text{T}}-[\text{A:P}] \right)}{[\text{A:P}]}$ | (12) |
| --- | --- | --- |

which can be solved for the unknown concentration of A:P,

|  | $\left[ \text{A:P} \right]=\frac{{[\text{A}]}_{\text{T}}+{[\text{P}]}_{\text{T}}+K_{\text{dAP1}}-\sqrt{\left( \text{[A]}_{\text{T}}+\text{[P]}_{\text{T}}+K_{\text{dAP1}} \right)^{2}-4\text{[A]}_{\text{T}}\text{[P]}_{\text{T}}}}{2}$ | (13) |
| --- | --- | --- |

Meanwhile, the total number of *Per* E-boxes, [E*_p_*]_T_ = [E*_p_*] + [A:E*_p_*] + [P:A:E*_p_*], can be written as:

|  | ${[\text{E}_{p}]}_{\text{T}}=\frac{K_{\text{dAE1}}[\text{A:}\text{E}_{p}]}{[\text{A}]}+\left[ \text{A:}\text{E}_{p} \right]+\frac{\text{[P][A:}\text{E}_{p}\text{]}}{K_{\text{dAP2}}}$ | (14) |
| --- | --- | --- |

which can be rearranged to give the probability that a *Per* gene is being transcribed:

|  | $\frac{[\text{A:}\text{E}_{p}]}{{[\text{E}_{p}]}_{\text{T}}}=\frac{[\text{A}]}{K_{\text{dAE1}}+\left[ \text{A} \right]+\frac{[\text{A][P}]}{K_{\text{dAP2}}}}$ | (15) |
| --- | --- | --- |

Plugging the definition of *K*_dAP1_ (Eq. (9)) into Eq. (15) yields

|  | $\frac{[\text{A:}\text{E}_{p}]}{{[\text{E}_{p}]}_{\text{T}}}=\frac{[\text{A}]}{K_{\text{dAE1}}+[\text{A}]+[\text{A:P}]\frac{K_{\text{dAP1}}}{K_{\text{dAP2}}}}$ | (16) |
| --- | --- | --- |

First Case. PER:CRY binds equally strongly to free- and E-box-bound BMAL1:CLOCK, i.e.,

|  | $K_{\text{dAP1}}=K_{\text{dAP2}}$ | (17) |
| --- | --- | --- |

In this case, Eq. (16) becomes

|  | $\frac{\left[ \text{A:}\text{E}_{p} \right]}{{[\text{E}_{p}]}_{\text{T}}}=\frac{[\text{A}]}{K_{\text{dAE1}}+\left[ \text{A} \right]+\left[ \text{A:P} \right]}$ | (18) |
| --- | --- | --- |

Plugging Eq. (3) into Eq. (18) yields Rate Law 1:

|  | $\frac{\left[ \text{A:}\text{E}_{p} \right]}{{[\text{E}_{p}]}_{\text{T}}}=\frac{[\text{A}]}{K_{\text{dAE1}}+\left[ \text{A} \right]_{\text{T}}}$ | (19) |
| --- | --- | --- |

Second Case. PER:CRY binds equally strongly to free- and E-box-bound BMAL1:CLOCK, Eq. (17) (First Case above), and at the same time, BMAL1:CLOCK saturates the *Per* E-box, i.e.,

|  | $K_{\text{dAE1}}\ll\left[ \text{A} \right]_{\text{T}}$ | (20) |
| --- | --- | --- |

In this case, Eq. (19) becomes Rate Law 0 in the original Kim-Forger model:

|  | $\frac{\left[ \text{A:}\text{E}_{p} \right]}{{[\text{E}_{p}]}_{\text{T}}}=\frac{[\text{A}]}{\left[ \text{A} \right]_{\text{T}}}$ | (21) |
| --- | --- | --- |

Third Case. BMAL1:CLOCK cannot or can hardly bind PER:CRY and E-box simultaneously, i.e.,

|  | $K_{\text{dAP2}}\gg K_{\text{dAP1}}$ | (22) |
| --- | --- | --- |

In this case, Eq. (16) gives rise to Rate Law 2:

|  | $\frac{\left[ \text{A:}\text{E}_{p} \right]}{{[\text{E}_{p}]}_{\text{T}}}=\frac{[\text{A}]}{K_{\text{dAE1}}+\left[ \text{A} \right]}$ | (23) |
| --- | --- | --- |

Electron microscopy studies by Aryal et al. have shown that PER:CRY::BMAL1:CLOCK complexes bind to E-boxes [3], indicating that rate laws 0 and 1 are to be preferred to rate law 2.

# References

1. Koike N, Yoo SH, Huang HC, Kumar V, Lee C, Kim TK, et al. Transcriptional architecture and chromatin landscape of the core circadian clock in mammals. Science. 2012;338(6105): 349-54. doi: 10.1126/science.1226339.

2. Narumi R, Shimizu Y, Ukai-Tadenuma M, Ode KL, Kanda GN, Shinohara Y, et al. Mass spectrometry-based absolute quantification reveals rhythmic variation of mouse circadian clock proteins. Proc Natl Acad Sci U S A. 2016;113(24): E3461-E7. doi: 10.1073/pnas.1603799113.

3. Aryal RP, Kwak PB, Tamayo AG, Gebert M, Chiu PL, Walz T, et al. Macromolecular Assemblies of the Mammalian Circadian Clock. Mol Cell. 2017;67(5): 770-82. doi: 10.1016/j.molcel.2017.07.017.
