# Supplementary material for: Mathematical analysis of robustness of oscillations in models of the mammalian circadian clock
Source: PLoS Comput Biol. 2022 Mar 18;18(3):e1008340. doi: 10.1371/journal.pcbi.1008340 (PMC8979472; doi:10.1371/journal.pcbi.1008340)
Supplement: S5 Text — (DOCX) [file pcbi.1008340.s010.docx]

# S5 Text. Non-dimensionalization of the modified Kim-Forger equations.

The models presented in this paper, as well as Kim and Forger’s original models, were cast in non-dimensional form before simulation and analysis. For example, we show how to non-dimensionalize the SNF(1M8) model, and the other variations use the same non-dimensionalization factors for the variables.

| Dimensional Equations | Non-dimensional Equations |
| --- | --- |
| $\frac{d\hat{M}}{d\hat{t}}=\hat{\alpha}_{1}\frac{\hat{A}_{\mathrm{free}}}{\hat{K}_{\text{A}}+\hat{A}_{T}}-\hat{\beta}_{1}\hat{M}$ | $\frac{dM}{dt}=\alpha\frac{A_{\mathrm{free}}}{K_{\text{A}}+A_{T}}-M$ |
| $\frac{d\hat{P}_{1}}{d\hat{t}}=\hat{\alpha}_{2}\hat{M}-\hat{\beta}_{1}\hat{P}_{1}$ | $\frac{dP_{1}}{dt}=M-P_{1}$ |
| $\frac{d\hat{P}_{2}}{d\hat{t}}=\hat{\alpha}_{3}\hat{P}_{1}-\hat{\beta}_{1}\hat{P}_{2}$ | $\frac{dP_{2}}{dt}=P_{1}-P_{2}$ |
| $\frac{d\hat{P}_{3}}{d\hat{t}}=\hat{\alpha}_{4}\hat{P}_{1}-\hat{\beta}_{1}\hat{P}_{3}$ | $\frac{dP_{3}}{dt}=P_{2}-P_{3}$ |
| $\frac{d\hat{P}_{4}}{d\hat{t}}=\hat{\alpha}_{5}\hat{P}_{3}-\hat{\beta}_{1}\hat{P}_{4}$ | $\frac{dP_{4}}{dt}=P_{3}-P_{4}$ |
| $\frac{d\hat{P}_{5}}{d\hat{t}}=\hat{\alpha}_{6}\hat{P}_{4}-\hat{\beta}_{1}\hat{P}_{5}$ | $\frac{dP_{5}}{dt}=P_{4}-P_{5}$ |
| $\frac{d\hat{P}_{6}}{d\hat{t}}=\hat{\alpha}_{7}\hat{P}_{5}-\hat{\beta}_{1}\hat{P}_{6}$ | $\frac{dP_{6}}{dt}=P_{5}-P_{6}$ |
| $\frac{d\hat{P}}{d\hat{t}}=\hat{\alpha}_{8}\hat{P}_{6}-\frac{\hat{\beta}_{\text{max}}\hat{P}}{\hat{K}_{\text{m}}+\hat{P}}$ | $\frac{dP}{dt}=P_{6}-\frac{\beta_{\text{max}}P}{K_{\text{m}}+P}$ |
| $2\hat{A}_{\mathrm{free}}=\hat{A}_{\text{T}}-\hat{P}-\hat{K}_{\text{d}}+\sqrt{\left( \hat{A}_{\text{T}}-\hat{P}-\hat{K}_{\text{d}} \right)^{2}+4\hat{A}_{\text{T}}\hat{K}_{\text{d}}}$ | $2A_{\mathrm{free}}=A_{\text{T}}-P-1+\sqrt{\left( A_{\text{T}}-P-1 \right)^{2}+4A_{\text{T}}}$ |

where we have already set $\hat{\beta}_{1}=\hat{\beta}_{2}\ldots{=\hat{\beta}}_{7}$, because this constraint makes oscillations most likely. Species P_1_ … P_6_ represent both mRNA species (say, P_1_ = mature mRNA in nucleus, P_2_ = mRNA in cytoplasm) and PER proteins in the cytoplasm (say, P_3_ = unphosphorylated PER, P_4_ = monophosphorylated PER, etc.), and P = nuclear PER. The purpose of non-dimensionalization is to ‘scale away’ as many of the kinetic parameters as possible, to reduce the dimensionality of the space of independent parameters. To this end, we make the following change of variables from ‘hat-wearing’ variables (carrying physical units) to their respective dimensionless versions:

$M=\frac{\hat{M}}{\hat{K}_{\text{d}}}\frac{\hat{\alpha}_{2}\hat{\alpha}_{3}{\cdot\cdot\cdot\hat{\alpha}}_{8}}{\hat{\beta}_{1}^{7}}$ , $P_{1}=\frac{\hat{P}_{1}}{\hat{K}_{\text{d}}}\frac{\hat{\alpha}_{3}\hat{\alpha}_{4}{\cdot\cdot\cdot\hat{\alpha}}_{8}}{\hat{\beta}_{1}^{6}}$ , $P_{2}=\frac{\hat{P}_{2}}{\hat{K}_{\text{d}}}\frac{\hat{\alpha}_{4}{\cdot\cdot\cdot\hat{\alpha}}_{8}}{\hat{\beta}_{1}^{5}}$ , …, $P_{6}=\frac{\hat{P}_{6}}{\hat{K}_{\text{d}}}\frac{\hat{\alpha}_{8}}{\hat{\beta}_{1}}$ , $P=\frac{\hat{P}}{\hat{K}_{\text{d}}}$ , ${t=\hat{\beta}}_{1}\hat{t}$

The dimensionless ODEs (above right) are governed by five dimensionless parameters:

$K_{\text{A}}=\frac{\hat{K}_{\text{A}}}{\hat{K}_{\text{d}}}$ , $K_{\text{m}}=\frac{\hat{K}_{\text{m}}}{\hat{K}_{\text{d}}}$ , $A_{\text{T}}=\frac{\hat{A}_{\text{T}}}{\hat{K}_{\text{d}}}$, $\beta_{\text{max}}=\frac{\hat{\beta}_{\text{max}}}{\hat{\beta}_{1}\hat{K}_{\text{d}}}$ , and $\alpha=\frac{\hat{\alpha}_{1}\hat{\alpha}_{2}{\cdot\cdot\cdot\hat{\alpha}}_{8}}{\hat{\beta}_{1}^{8}\hat{K}_{\text{d}}}$ .
