# Supplementary material for: Mathematical analysis of robustness of oscillations in models of the mammalian circadian clock
Source: PLoS Comput Biol. 2022 Mar 18;18(3):e1008340. doi: 10.1371/journal.pcbi.1008340 (PMC8979472; doi:10.1371/journal.pcbi.1008340)
Supplement: S1 Code — (DOCX) [file pcbi.1008340.s011.docx]

**SUPPLEMENTARY MATERIAL: Computer Codes**

X. Yao, B.L. Heidebrecht, J. Chen & J.J. Tyson

**ODE files for use with XPP-Auto**

**# SNF(0L3) model (Fig 3b)**

Afree = 0.5*(AT-Pn-Kd + sqrt((AT-Pn-Kd)^2+4*Kd*AT))

dM/dt = alf*Afree/AT - M

dPtot/dt = M - Pn

dPn/dt = Ptot - 2*Pn

aux Pc=Ptot-Pn

par AT=1000, alf=20000, Kd=1

@ Yplot=M, Ylo=0, Yhi=.2

done

**# SNF(0L8) model (Fig 4c)**

Afree = 0.5*(At-Pn-Kd + sqrt((At-Pn-Kd)^2+4*Kd*AT))

dm/dt = alf*Afree/AT - m

dp0/dt = m - p0

dp1/dt = p0 - p1

dp2/dt = p1 - p2

dp3/dt = p2 - p3

dp4/dt = p3 - p4

dp5/dt = p4 - p5

dpn/dt = p5 - pn

aux ptot = (p0+p1+p2+p3+p4+p5+pn)

par AT=40, alf=200, Kd=1

@ Yplot=m, Ylo=0, Yhi=5

done

**# SNF(0M8) model (Fig 5b)**

Afree = 0.5*(AT-Pn-Kd + sqrt((AT-Pn-Kd)^2+4*Kd*AT))

dm/dt = alf*Afree/AT - m

dp0/dt = m - p0

dp1/dt = p0 - p1

dp2/dt = p1 - p2

dp3/dt = p2 - p3

dp4/dt = p3 - p4

dp5/dt = p4 - p5

dPn/dt = p5 - Bmax*Pn/(Km+Pn)

aux ptot = (p0+p1+p2+p3+p4+p5+Pn)

par alf=20, AT=16

par Bmax=3.8, Km=1

par Kd=1

@ TOTAL=100, Xlo=0, Xhi=100, Yplot=Ptot, Ylo=0, Yhi=5

done

**# SNF(1M8) model (Fig 7b)**

Afree = 0.5*(AT-Pn-Kd + sqrt((AT-Pn-Kd)^2+4*Kd*AT))

dm/dt = alf*Afree/(KA+AT) - m

dp0/dt = m - p0

dp1/dt = p0 - p1

dp2/dt = p1 - p2

dp3/dt = p2 - p3

dp4/dt = p3 - p4

dp5/dt = p4 - p5

dPn/dt = p5 - Bmax*Pn/(Km+Pn)

aux ptot = (p0+p1+p2+p3+p4+p5+Pn)

par alf=50, AT=20, Bmax=5

par Kd=1, Km=5.5, KA=20

@ TOTAL=100, Xlo=0, Xhi=100, Yplot=Ptot, Ylo=0, Yhi=5

done

**# NNF(1M8) model (Fig 8b)**

Afree = 0.5*(AT-Pn-Kd + sqrt((AT-Pn-Kd)^2+4*Kd*AT))

RL1 = Afree/(KA+AT)

dm/dt = alf*RL1 - m

dp0/dt = m - p0

dp1/dt = p0 - p1

dp2/dt = p1 - p2

dp3/dt = p2 - p3

dp4/dt = p3 - p4

dp5/dt = p4 - p5

dPn/dt = p5 - Bmax*Pn/(Km+Pn)

dAT/dt = del*(Amax/(1+V) - AT)

dV/dt = del*(Vmax*RL1 - V)

aux ptot = (p0+p1+p2+p3+p4+p5+Pn)

par alf=30, Amax=30, Vmax=22, Bmax=4.5

par Kd=1, Km=2.5, KA=3.7, del=0.17

@ TOTAL=100, Xlo=0, Xhi=100, Yplot=Ptot, Ylo=0, Yhi=5

done

**# PNF(1M8) model (Fig 9b)**

Afree = 0.5*(AT-Pn-Kd + sqrt((AT-Pn-Kd)^2+4*Kd*AT))

RL1 = Afree/(KA+AT)

dm/dt = alf*RL1 - m

dp0/dt = m - p0

dp1/dt = p0 - p1

dp2/dt = p1 - p2

dp3/dt = p2 - p3

dp4/dt = p3 - p4

dp5/dt = p4 - p5

dPn/dt = p5 - Bmax*Pn/(Km+Pn)

dAT/dt = del*(Amax*(eps+R)/(1+R) - AT)

dR/dt = del*(Rmax*RL1 - R)

aux ptot = (p0+p1+p2+p3+p4+p5+Pn)

par alf=10, Amax=15, Rmax=6.2, Bmax=1.85

par Kd=1, Km=8.5, KA=35, del=8, eps=0.0003

@ TOTAL=100, Xlo=0, Xhi=100, Yplot=Ptot, Ylo=0, Yhi=5

done
